# Synthesis, Insecticidal, Fungicidal Activities and Structure–Activity Relationships of Tschimganin Analogs

**DOI:** 10.3390/molecules23061473

**Published:** 2018-06-18

**Authors:** Yueting Zhou, Chunjuan Wang, Fang Xin, Xiaoqiang Han, Jie Zhang, Ke Sun

**Affiliations:** 1 Key Laboratory at Universities of Xinjiang Uygur Autonomous Region for Oasis Agricultural Pest Management and Plant Protection Resource Utilization, College of Agricultural, Shihezi University, 221st Beisi Road, Shihezi 832002, China; zhouyueting@stu.shzu.edu.cn (Y.Z.); wchunjuan@126.com (C.W.); xinf@stu.shzu.edu.cn (F.X.); 2 Key Laboratory for Green Processing of Chemical Engineering of Xinjiang Production and Construction Group, School of Chemistry and Chemical Engineering, 221st Beisi Road, Shihezi 832003, China; 3 State Key Laboratory of the Discovery and Development of Novel Pesticide, Shenyang Sinochem Agrochemicals R&D Co., Ltd., No. 8-1 Shenliao Dong Road, Tiexi District, Shenyang 110021, China; sunke@sinochem.com

**Keywords:** tschimganin analogs, insecticidal activities, fungicidal activity, structure-activity relationship (SAR)

## Abstract

For the first time, a novel series of tschimganin analogs were designed, synthesized, and evaluated for their insecticidal and fungicidal activities. Their structures were characterized by ^1^H-NMR, ^13^C-NMR and HRMS. Some of these compounds displayed excellent insecticidal and fungicidal activities, suggesting that they have potential to be used as bifunctional agrochemicals. Compound **3d** and **3g** with electron donating groups showed better inhibitory activity and growth inhibition activity towards *Helicoverpa armigera* (Hübner). The properties and positions of the substituents on the benzene ring have an important influence on the acaricidal activity of tschimganin analogs. Topomer comparative molecular field analysis (CoMFA) was employed to develop a three-dimensional quantitative structure-activity relationship model for the compounds against *Tetranychus turkestani* Ugarov et Nikolski. It was indicated that higher electronegativity was beneficial for acaricidal activity. Moreover, compound **3r** having a 2-hydroxy-3,5- dinitrophenyl moiety displayed a fungicidal spectrum as broad as azoxystrobin against these phytopathogens.

## 1. Introduction

**Figure 1:**
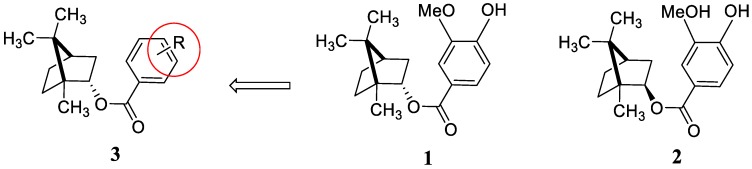
Design strategy for novel tertiary alcohols.

Botanical pesticides refer to plants, crude plant extracts or the derivatives of the active ingredients used for the protection of crops and stored products from insect pests. These can be recommended as an ecochemical and sustainable strategy for the management of agricultural pests due to their biodegradable nature, systemicity after application, capacity to alter the behavior of target pests, and their favorable safety profile [1]. The exclusively Old-World genus *Ferula* belongs to the family Umbelliferae, with about 150 species distributed throughout the Mediterranean area and central Asia, especially in the former USSR, Iran and northwest China (e.g., Xinjiang Province) [2]. Some plants of the genus *Ferula* have been used as pharmaceutical plants in many countries for centuries [3]. *Ferula* is a rich source of biologically active compounds, such as coumarin derivatives, sesquiterpenes, aromatic lactones, and disulfide compounds [4]. Plant extracts and metabolites from the *Ferula* genus were reported to possess many biological features, including antiviral [5], anti-inflammatory [6], antitumor [7,8], anticancer [9], antiulcerative [10], antidiabetic [11], antibacterial [12,13], acaricidal [14], antiprotozoal, and antiglycation [15] activities. The diastereomeric monoterpene benzoates tschimganin (**1**) and isotschimganin (**2**) were isolated from *Ferula dissecta* (Figure 1). The growth inhibition effects of **1** and **2** exhibited different degrees of cytotoxic effects against HeLa cells at 24 h after treatment with IC_50_ values of 32.1 and 29.1 μM, respectively [16]. Tschimganin (**1**) was also found to be a novel drug resistance reversal agent for *Staphylococcus aureus* [17]. The pooled results indicate that tschimganin (**1**) has good antibacterial and anti-tumor activities. However, the synthesis and structure-activity relationship of tschimganin analogs have not been reported. Inspired by these results, we were encouraged to further investigate the effect on fungicidal and insecticidal activities by tschimganin. Therefore, in this report, we systematically designed and synthesized a novel series of tschimganin analogs (**3**) and evaluated them for insecticidal and fungicidal activities. Their structure-activity relationship (SAR) also was studied.

## 2. Results and Discussion

**Scheme 1:**
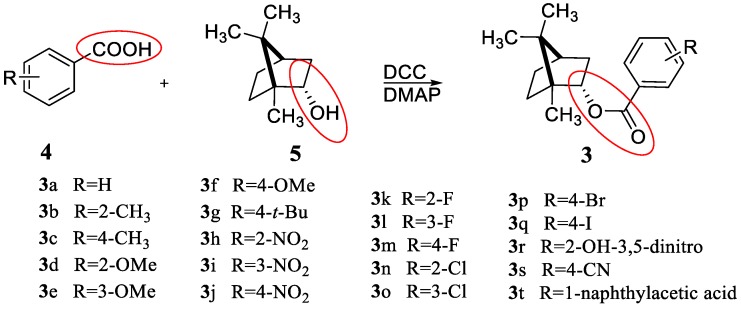
Synthetic route for tschimganin analogs **3**.

The synthesis of tschimganin analogs **3**, which features a catalytic esterification, is shown in Scheme 1. The tschimganin analogs **3** were prepared in excellent yield from commercially available substituted benzoic acids **4** and 1*S*,2*S*,4*R*-fenchyl alcohol (**5**) via room temperature esterification catalyzed by 4-dimethylaminopyridine (DMAP) and dicyclohexylcarbodiimide (DCC).

**Figure 2:**
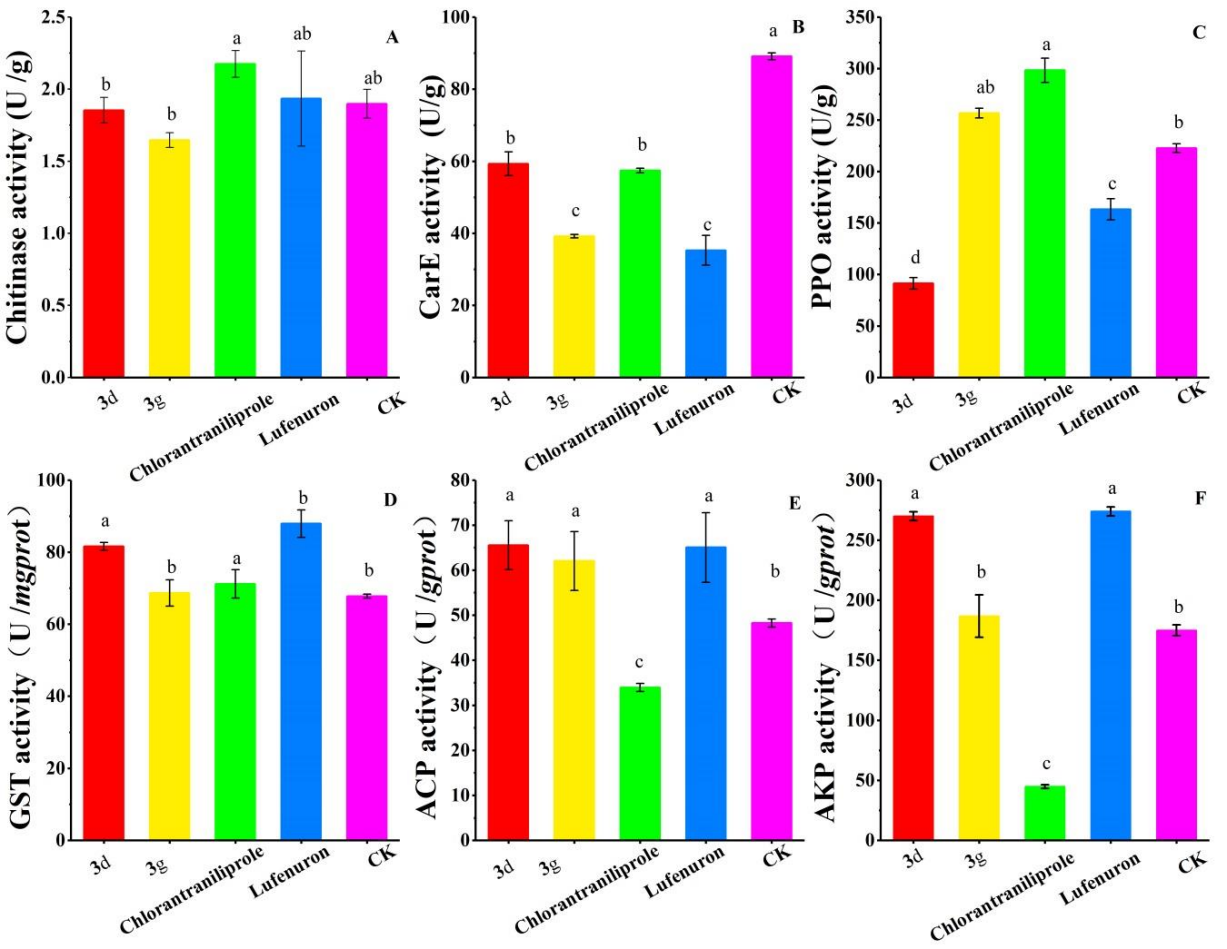
Effect on *H. armigera* (Hübner) enzyme activity by tschimganin analogs. (**A**) Chitinase activity; (**B**) CarE activity; (**C**) PPO activity; (**D**) GST activity; (**E**) ACP activity; (**F**) AKP activity. ^a–c^ (*p* < 0.05; Duncan’s test).

To characterize the biological potential of these tschimganin analogs **3**, the insecticidal activities of all the new compounds were tested against *Helicoverpa armigera* (Hübner), *Tetranychus truncates* and *Tetranychus turkestani* Ugarov et Nikolski, and their in vitro antifungal activities were evaluated against seven plant pathogenic fungi. The commercial insecticides chlorantraniliprole, biflenazate, pyridaben, hexythiazox and commercial fungicides azoxystrobin, kresoxim-methyl, trifloxystrobin were used as control. As shown in Table 1, the tschimganin analogs **3** exhibited moderate or weak activity to *H. armigera* (Hübner). Only the compounds **3d**, **3e**, **3f**, and **3g** containing electron-donating groups showed medium insecticidal activity. Compound **3g** showed the best activity (60%), which implied that the electron-donating groups (alkyl and alkoxy) were beneficial to the insecticidal activity. Interestingly, most of the tschimganin analogs displayed *H. armigera* (Hübner) growth inhibition activity. Therefore, we further studied their effect on the metabolic enzyme activity of bollworm (Figure 2).

The target compounds showed different inhibitory activities on cotton bollworm metabolic enzymes. Compound **3d** and lufenuron showed similar effects on the six enzyme activities: they displayed no activity on chitinase, inhibition of carboxylesterase (CarE) and polyphenol oxidase (PPO), and activation of glutathione *S*-transferase (GST) and phosphatases (acid phosphatase, ACP and alkaline phosphatase, AKP). Compound **3g** and chlorantraniliprole showed significant activation of PPO activity, and no effects on GST activity. In contrast **3g** displayed a slight inhibition of chitinase while chlorantraniliprole showed activation of chitinase. Meanwhile, chlorantraniliprole displayed a significant inhibition of phosphatases (ACP and AKP). Inspired by the tschimganin analogs *H. armigera* (Hübner) activity, we were encouraged to further investigate the effects on the cotton spider mite.

The foliar contact activities of the synthesized compounds against two cotton spider mite species ae shown in Table 1, in comparison to biflenazate, pyridaben and hexythiazox. In general, all of the compounds displayed considerable to excellent acaricidal activity against the two kinds of cotton spider mite. Tschimganin analogs with 4-*t*-Bu (**3g**) and 4-F (**3m**) at the benzene ring exhibited activity against *T. truncates* similar to pyridaben. Compound **3i**, with 3-NO_2_ in the benzene ring, exhibited much better activity than pyridaben, and activity similar to biflenazate and hexythiazox. Notably, tschimganin analogs with 4-OMe (**3f**), 2-NO_2_ (**3h**), 3-NO_2_ (**3i**) and 4-NO_2_ (**3j**) at the benzene ring displayed activity similar to biflenazate and hexythiazox and slightly lower than that of pymetrozine against *T. turkestani* Ugarov et Nikolski (Table 1).

However, tschimganin analogs with alkyl, Cl, Br, I, NH_2_, CN and naphthylacetic acid on the benzene ring exhibited much lower activity than control against the two cotton spider mite species. The results indicated that the additive effect of the *t*-Bu and NO_2_ group play an important role in acaricidal activity. In addition, the properties and positions of these substituents on the benzene ring have an important influence on the acaricidal activity of these compounds. Compound **3i**, having a NO_2_ group at the *meta* position of the benzene ring, exhibited good activity against both cotton spider mite species. **3h** and **3j** only exhibited activity against *T. turkestani* Ugarov et Nikolski; they have a NO_2_ group at the *ortho* and para positions of the benzene ring, respectively.

**Figure 3:**
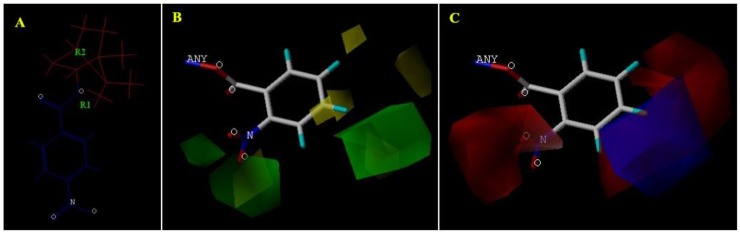
(**A**) Topomer comparative molecular field analysis (CoMFA) contour maps of **3j**; (**B**) The steric field around R1 of **3j**; (**C**) The electrostatic field around R1 of **3j**.

The topomer comparative molecular field analysis (CoMFA) model was optimized. A cross-validation *q*^2^ value of 0.511 and a non-cross-validation *r*^2^ value of 0.830 with an optimized component of 3 were obtained, which suggested that the model has good predictive ability (*q*^2^ > 0.5). The sterically favored and disfavored regions are shown in green and yellow. In the electrostatic field, the positively charged favored regions are shown in blue, and the negatively charged favored regions are shown in red. We chose the molecule **3j**, which had the highest activity against *T. turkestani* Ugarov et Nikolski; this makes it is easier to explain the contour map. The activity of the compound is affected by the interaction between the three-dimensional field and the electrostatic field. Compounds **3g**, **3h**, **3i**, and **3j** all have large-volume groups (Figure 3B). However, there are large red regions on both sides of the benzene ring (Figure 3C), indicating that electronegativity was beneficial for acaricidal activity. Therefore, the nitro-substituted compounds (**3h**, **3i**, and **3j**) had higher activity than the *tert*-butyl-substituted compound (**3g**).

Compounds **3a**–**3w** were evaluated in a series of fungicidal tests in vitro, against a range of phytopathogenic species. The resulting data (Table 2) revealed that these tschimganin analogs displayed potential fungicidal activity against kinds of plant fungi, including *Colleetotrichum lagenarium* (Pass.) Ell. et Halst, *Rhizoctonia solani, Fulvia fulva* (Cooke) Cif., *Pyricularia grisea* (Cooke) Sacc., *Alternaria alternata* Japanese pear pathotype, sunflower sclerotinia rot, and rape sclerotinia rot.

In general, these compounds were highly active against sunflower sclerotinia rot and rape sclerotinia rot, and possessed poor activity against *C. lagenarium* (Pass.) Ell. et Halst and *R. solani* in vitro. Similar to acaricidal activity, the properties of these substituents on the benzene ring have an important influence on the fungicidal activities of these compounds. Compound **3m**, which have electron-withdrawing group F atom, showed moderate fungicidal activity for sunflower sclerotinia rot, rape sclerotinia rot, *F. fulva* (Cooke) Cif., and *A. alternata* Japanese pear pathotype. 

Compound **3e**, having a methoxy at the *meta* position of the benzene ring, exhibited good activity against *F. fulva* (Cooke) Cif., *P. grisea* (Cooke) Sacc., *A. alternata* Japanese pear pathotype and sunflower sclerotinia rot. Compound **3q**, possessing an iodine substituent at the *para* position of the benzene ring, exhibited good activity against *A. alternata* Japanese pear pathotype, sunflower sclerotinia rot, and rape sclerotinia rot. Notably, compound **3r** having a 2-hydroxy-3,5-dinitrophenyl moiety displayed a fungicidal spectrum as broad as azoxystrobin against these phytopathogens. We didn’t find an explicit relationship between their structures and fungicidal activities for mycelium inhibition.

## 3. Materials and Methods

### 3.1. Materials and Measurements

^1^H-NMR and ^13^C-NMR spectra were obtained at 400/101 MHz using an Avance III HD 400 spectrometer (Bruker, Zurich, Switzerland) in CDCl_3_ solution with TMS as the internal standard. Chemical shift values (δ) were given in parts per million. HRMS data were obtained on a LTQ Orbitrap XL Discovery (Thermo Scientific, Bremen, Germany) instrument. Commercially available compounds were used in this work without further purification. The solvent tetrahydrofuran (THF) and benzene was dried by distillation from sodium and benzophenone. The CH_2_Cl_2_ (DCM) was dried by distillation CaH_2_. Equipment used included a HVE-50 autoclave (Hirayama Manufacturing Corporation, Tokyo, Japan), a A1000IN Intelligent artificial climate chamber (Conviron, Winnipeg, Manitoba Canada), a DL-CJ-1ND Super Clear Workbanch (Donglian Elactronic & Technology Development Co., Ltd., Beijing, China). Potato dextrose agar was purchased from Qingdao Hope Bio-Technogy Co., Ltd. (Qingdao, China). Chlorantraniliprole, lufenuron, biflenazate, pyridaben and hexythiazox standards were purchased from J&K Scientific Ltd. (Beijing, China). Enzyme activity kits were purchased from Nanjing Jiancheng Bioengineering Institute, (Nanjing, China). *H. armigera* (Hübner), *T. truncates*, *T. turkestani* Ugarov et Nikolski were reared in lab. Plant pathogens *C. lagenarium* (Pass.) Ell. et Halst, *R. solani*, *F. fulva* (Cooke) Cif., *P. grisea* (Cooke) Sacc., *A. alternata* Japanese pear pathotype, sunflower sclerotinia rot and rape sclerotinia rot were provided by the Key Laboratory at Universities of Xinjiang Uygur Autonomous Region for Oasis Agricultural Pest Management and Plant Protection Resource Utilization Shihezi, China).

### 3.2. General Synthetic Method for Compounds ***3***

A mixture of substituted benzoic acid **4** (0.01 mol), 1*S*,2*S*,4*R*-fenchyl alcohol (**5**, 0.011 mol), 4-dimethylaminopyridine (DMAP, 0.002 mol), and dichloromethane (DCM, 25 mL) was stirred at 0 °C. Then, *N*,*N*′-dicyclohexylcarbodiimide (DCC, 0.008 mol) dissolved in DCM (5 mL) was added dropwise at 0 °C and then stirred for 10 h at room temperature. The mixture was filtered, and the filtrate concentrated under reduced pressure. The residue was subjected to silica gel chromatography using PE and EtOAc as eluent to afford the appropriate compound **3**.

*(1S,2S,4R)-1,7,7-Trimethylbicyclo[2.2.1]heptan-2-yl benzoate* (**3a**): colorless oil, yield 82%. ^1^H-NMR δ 8.14–8.04 (m, 2H), 7.64–7.54 (m, 1H), 7.51–7.43 (m, 2H), 4.65 (d, *J* = 1.9 Hz, 1H), 2.02–1.92 (m, 1H), 1.85–1.77 (m, 2H), 1.72–1.67 (m, 1H), 1.59–1.49 (m, 1H), 1.28 (dd, *J* = 10.3, 1.5 Hz, 1H), 1.21 (s, 3H), 1.14 (s, 3H), 0.87 (s, 3H). ^13^C-NMR δ 166.88, 132.76, 130.73, 86.68, 48.65, 48.45, 41.49, 29.78, 26.91, 25.94, 20.33, 19.52. HRMS (*m*/*z*) calcd. for C_17_H_23_O_2_^+^ (M + H)^+^ 259.16926, found 259.16925.

*(1S,2S,4R)-1,7,7-Trimethylbicyclo[2.2.1]heptan-2-yl 2-methylbenzoate* (**3b**): white powder, m.p. 41.5–42.5 °C, yield 83%. ^1^H-NMR δ 7.95 (dd, *J* = 8.1, 1.2 Hz, 1H), 7.40 (td, *J =* 7.5, 1.4 Hz, 1H), 7.26 (q, *J =* 5.2 Hz, 2H), 4.60 (d, *J =* 1.9 Hz, 1H), 2.62 (s, 3H), 1.94–1.85 (m, 1H), 1.80–1.73 (m, 2H), 1.67 (dd, *J =* 10.3, 1.7 Hz, 1H), 1.54–1.46 (m, 1H), 1.24 (dd, *J =* 10.3, 1.4 Hz, 1H), 1.21 (s, 3H), 1.16 (ddd, *J =* 6.2, 3.3, 2.1 Hz, 1H), 1.13 (d, *J =* 4.8 Hz, 3H), 0.86 (s, 3H). ^13^C-NMR δ 168.01, 140.08, 131.74, 131.67, 130.48, 130.23, 125.70, 86.92, 48.52, 48.48, 41.50, 39.75, 29.76, 27.03, 25.91, 21.93, 20.44, 19.60. HRMS (*m*/*z*) calcd. for C_18_H_25_O_2_^+^ (M + H)^+^ 273.18491, found 273.18500.

*(1S,2S,4R)-1,7,7-Trimethylbicyclo[2.2.1]heptan-2-yl 4-methylbenzoate* (**3c**): colorless oil, yield 86%. ^1^H-NMR δ 8.01–7.90 (m, 2H), 7.24 (s, 2H), 4.61 (d, *J =* 1.9 Hz, 1H), 2.42 (s, 3H), 1.98–1.90 (m, 1H), 1.82–1.74 (m, 2H), 1.69–1.64 (m, 1H), 1.50 (ddd, *J =* 12.6, 5.8, 4.1 Hz, 1H), 1.28–1.20 (m, 2H), 1.18 (s, 3H), 1.10 (s, 3H), 0.84 (s, 3H). ^13^C-NMR δ 171.96, 133.82, 132.16, 131.01, 128.68, 128.00, 127.94, 126.21, 125.72, 125.44, 124.04, 86.74, 48.30, 48.28, 41.28, 39.46, 39.40, 29.60, 26.41, 25.71, 19.94, 19.26. HRMS (*m*/*z*) calcd. for C_18_H_25_O_2_^+^ (M + H)^+^ 273.18491, found 273.18494.

*(1S,2S,4R)-1,7,7-Trimethylbicyclo[2.2.1]heptan-2-yl 2-methoxybenzoate* (**3d**): light yellow oil, yield 89%. ^1^H-NMR δ 7.86 (dd, *J =* 7.9, 1.8 Hz, 1H), 7.47 (ddd, *J =* 9.1, 7.5, 1.8 Hz, 1H), 7.02–6.95 (m, 2H), 4.59 (d, *J =* 1.9 Hz, 1H), 3.91 (s, 3H), 1.96–1.87 (m, 1H), 1.76 (ddq, *J =* 9.1, 6.1, 3.0 Hz, 2H), 1.67–1.62 (m, 1H), 1.49 (tdd, *J =* 12.5, 5.7, 4.1 Hz, 1H), 1.23 (dd, *J =* 10.3, 1.4 Hz, 1H), 1.19 (s, 3H), 1.15 (ddd, *J =* 12.4, 3.4, 2.1 Hz, 1H), 0.87 (s, 3H). ^13^C-NMR δ 166.49, 159.41, 133.35, 131.67, 120.45, 120.03, 111.98, 86.76, 55.84, 48.53, 41.45, 39.77, 29.69, 26.89, 25.90, 20.31, 19.52. HRMS (*m*/*z*) calcd. for C_18_H_25_O_3_^+^ (M + H)^+^ 289.17982, found 289.17987.

*(1S,2S,4R)-1,7,7-Trimethylbicyclo[2.2.1]heptan-2-yl 3-methoxybenzoate* (**3e**): colorless oil, yield 90%. ^1^H-NMR δ 7.66 (dd, *J =* 7.6, 0.9 Hz, 1H), 7.60 (dd, *J =* 2.4, 1.4 Hz, 1H), 7.36 (t, *J =* 7.9 Hz, 1H), 7.14–7.07 (m, 1H), 4.61 (d, *J =* 1.7 Hz, 1H), 3.86 (s, 3H), 2.00–1.86 (m, 1H), 1.82–1.74 (m, 2H), 1.69–1.65 (m, 1H), 1.56–1.48 (m, 1H), 1.25 (d, *J =* 10.3 Hz, 2H), 1.19 (s, 3H), 1.11 (s, 3H), 0.85 (s, 3H). ^13^C-NMR δ 166.76, 159.56, 132.04, 129.39, 121.88, 119.01, 114.27, 86.81, 55.40, 48.64, 48.44, 41.47, 39.86, 29.76, 26.90, 20.31, 19.51. HRMS (*m*/*z*) calcd. for C_18_H_25_O_3_^+^ (M + H)^+^ 289.17982, found 289.17984.

*(1S,2S,4R)-1,7,7-Trimethylbicyclo[2.2.1]heptan-2-yl 4-methoxybenzoate* (**3f**): colorless oil, yield 86%. ^1^H-NMR δ 8.05–8.00 (m, 2H), 6.96–6.92 (m, 2H), 4.59 (d, *J* = 1.9 Hz, 1H), 3.87 (s, 3H), 1.93 (tdd, *J* = 10.3, 6.1, 2.9 Hz, 1H), 1.81–1.74 (m, 2H), 1.66 (dt, *J* = 10.3, 2.2 Hz, 1H), 1.56 (s, 1H), 1.54–1.47 (m, 1H), 1.24 (dd, *J =* 10.3, 1.6 Hz, 1H), 1.18 (s, 3H), 1.10 (s, 3H), 0.84 (s, 3H). ^13^C-NMR δ 166.65, 163.23, 131.51, 123.18, 113.61, 86.29, 55.44, 48.62, 48.44, 41.48, 39.83, 29.76, 26.92, 25.94, 20.31, 19.52. HRMS (*m*/*z*) calcd. for C_18_H_25_O_3_^+^ (M + H)^+^ 289.17982, found 289.17981.

*(1S,2S,4R)-1,7,7-Trimethylbicyclo[2.2.1]heptan-2-yl 4-(tert-butyl)benzoate* (**3g**): white solid, m.p. 74.5–76.3 °C, yield 74%. ^1^H-NMR δ 8.06–8.02 (m, 2H), 7.52–7.48 (m, 2H), 4.64 (d, *J* = 1.9 Hz, 1H), 2.02–1.93 (m, 1H), 1.85–1.77 (m, 2H), 1.69 (dd, *J* = 10.3, 1.7 Hz, 1H), 1.59–1.51 (m, 1H), 1.37 (s, 9H), 1.27 (dd, *J =* 10.4, 1.5 Hz, 1H), 1.21 (s, 3H), 1.13 (s, 3H), 0.87 (s, 3H). ^13^C-NMR δ 166.87, 156.38, 129.44, 127.97, 125.35, 86.42, 48.64, 48.46, 41.49, 39.84, 35.07, 31.16, 29.78, 26.91, 25.95, 20.33, 19.51. HRMS (*m*/*z*) calcd. for C_21_H_31_O_2_^+^ (M + H)^+^ 315.23186, found 315.23184.

*(1S,2S,4R)-1,7,7-Trimethylbicyclo[2.2.1]heptan-2-yl 2-nitrobenzoate* (**3h**): white solid, m.p. 104.3–106.2 °C, yield 85%. ^1^H-NMR δ 7.89–7.84 (m, 1H), 7.82–7.78 (m, 1H), 7.66 (dqd, *J* = 15.0, 7.5, 1.6 Hz, 2H), 4.64 (d, *J =* 2.0 Hz, 1H), 1.80–1.76 (m, 1H), 1.73–1.63 (m, 3H), 1.53–1.43 (m, 1H), 1.25 (dd, *J* = 10.3, 1.5 Hz, 1H), 1.21 (s, 3H), 1.15 (s, 3H), 0.87 (s, 3H). ^13^C-NMR δ 165.53, 148.44, 132.58, 131.76, 130.05, 127.61, 123.70, 88.93, 48.65, 48.33, 41.49, 39.93, 29.66, 26.61, 25.78, 20.21, 19.41. HRMS (*m*/*z*) calcd. for C_17_H_22_NO_4_^+^ (M + H)^+^ 304.15433, found 304.15475.

*(1S,2S,4R)-1,7,7-Trimethylbicyclo[2.2.1]heptan-2-yl 3-nitrobenzoate* (**3i**): white powder, m.p. 84.0–85.0 °C, yield 91%. ^1^H-NMR δ 8.88 (dd, *J* = 2.8, 1.1 Hz, 1H), 8.48–8.37 (m, 2H), 7.73–7.66 (m, 1H), 4.68 (d, *J =* 1.9 Hz, 1H), 1.99–1.88 (m, 1H), 1.82 (tq, *J* = 9.0, 2.9 Hz, 2H), 1.70 (ddd, *J* = 10.4, 4.0, 2.3 Hz, 1H), 1.64–1.51 (m, 1H), 1.33–1.28 (m, 1H), 1.21 (s, 3H), 1.14 (s, 3H), 0.87 (s, 3H). ^13^C-NMR δ 164.76, 148.34, 135.15, 132.42, 129.67, 127.26, 124.43, 87.88, 48.63, 48.35, 41.44, 39.88, 29.73, 26.87, 25.89, 20.29, 19.48. HRMS (*m*/*z*) calcd. for C_17_H_22_NO_4_^+^ (M + H)^+^ 304.15433, found 304.15439.

*(1S,2S,4R)-1,7,7-Trimethylbicyclo[2.2.1]heptan-2-yl 4-nitrobenzoate* (**3j**): light yellow solid, m.p. 106.5–108.5 °C, yield 87%. ^1^H-NMR δ 8.35–8.29 (m, 2H), 8.27–8.21 (m, 2H), 4.67 (d, *J* = 1.9 Hz, 1H), 1.97–1.86 (m, 1H), 1.86–1.76 (m, 2H), 1.70 (ddd, *J* = 10.4, 4.0, 2.3 Hz, 1H), 1.63–1.51 (m, 1H), 1.30 (dd, *J* = 10.5, 1.5 Hz, 1H), 1.21 (s, 3H), 1.14 (s, 3H), 0.86 (s, 3H). ^13^C-NMR δ 164.95, 150.46, 136.02, 130.58, 123.57, 87.86, 48.64, 48.35, 41.42, 39.89, 29.72, 26.86, 25.87, 20.27, 19.46. HRMS (*m*/*z*) calcd. for C_17_H_22_NO_4_^+^ (M + H)^+^ 304.15433, found 304.15427.

*(1S,2S,4R)-1,7,7-Trimethylbicyclo[2.2.1]heptan-2-yl 2-fluorobenzoate* (**3k**)*:* colorless oil, yield 76%. ^1^H-NMR δ 7.97 (td, *J* = 7.6, 1.8 Hz, 1H), 7.51 (dddd, *J =* 8.3, 7.0, 4.8, 1.9 Hz, 1H), 7.21 (td, *J* = 7.7, 1.0 Hz, 1H), 7.14 (ddd, *J* = 10.8, 8.3, 0.9 Hz, 1H), 4.63 (d, *J* = 1.8 Hz, 1H), 1.98–1.89 (m, 1H), 1.77 (dd, *J =* 12.4, 6.0 Hz, 2H), 1.69–1.63 (m, 1H), 1.55–1.45 (m, 1H), 1.25 (dd, *J* = 10.3, 1.4 Hz, 1H), 1.19 (s, 3H), 1.12 (s, 3H), 0.86 (s, 3H). ^13^C-NMR δ 164.89, 163.33, 160.74, 134.25, 132.13, 123.92, 119.13, 117.14, 116.92), 87.57, 48.52, 48.47, 41.43, 39.76, 29.72, 26.83, 25.87, 20.27, 19.46. HRMS (*m*/*z*) calcd. for C_17_H_22_FO_2_^+^ (M + H)^+^ 277.15983, found 277.15973.

*(1S,2S,4R)-1,7,7-Trimethylbicyclo[2.2.1]heptan-2-yl 3-fluorobenzoate* (**3l**): colorless oil, yield 77%. ^1^H-NMR δ 7.69–7.64 (m, 1H), 7.60 (dd, *J* = 2.5, 1.5 Hz, 1H), 7.36 (t, *J* = 7.9 Hz, 1H), 7.10 (ddd, *J =* 8.3, 2.7, 0.9 Hz, 1H), 4.61 (d, *J =* 1.9 Hz, 1H), 1.98–1.89 (m, 1H), 1.82–1.74 (m, 2H), 1.69–1.64 (m, 1H), 1.52 (tdd, *J* = 12.5, 5.8, 4.1 Hz, 1H), 1.30–1.19 (m, 2H), 1.19 (s, 3H), 1.11 (s, 3H), 0.85 (s, 3H). ^13^C-NMR δ 166.78, 159.58, 132.05, 129.41, 121.89, 119.02, 114.28, 86.82, 55.41, 48.64, 48.44, 41.47, 39.86, 29.77, 26.91, 25.93, 20.32, 19.51. HRMS (*m*/*z*) calcd. for C_17_H_22_FO_2_^+^ (M + H)^+^ 277.15983, found 277.15985.

*(1S,2S,4R)-1,7,7-Trimethylbicyclo[2.2.1]heptan-2-yl 4-fluorobenzoate* (**3m**): colorless viscous liquid, yield 74%. ^1^H-NMR δ 8.14–8.05 (m, 2H), 7.18–7.11 (m, 2H), 4.63 (d, *J* = 1.9 Hz, 1H), 1.98–1.88 (m, 1H), 1.80 (tt, *J =* 9.1, 3.0 Hz, 2H), 1.71–1.66 (m, 1H), 1.54 (ddt, *J* = 12.6, 5.7, 4.0 Hz, 1H), 1.28 (dd, *J =* 10.4, 1.5 Hz, 1H), 1.20 (s, 3H), 1.13 (s, 3H), 0.86 (s, 3H). ^13^C-NMR δ 166.93, 165.90, 164.41, 132.04, 131.95, 126.95, 126.92, 115.59, 115.37, 86.84, 48.61, 48.40, 41.45, 39.83, 29.74, 26.89, 25.91, 20.29, 19.49. HRMS (*m*/*z*) calcd. for C_17_H_22_FO_2_^+^ (M + H)^+^ 277.15983, found 277.15979.

*(1S,2S,4R)-1,7,7-Trimethylbicyclo[2.2.1]heptan-2-yl 2-chlorobenzoate* (**3n**): white solid, m.p. 63.1–63.4 °C, yield 78%. ^1^H-NMR δ 7.90–7.85 (m, 1H), 7.50–7.40 (m, 2H), 7.37–7.31 (m, 1H), 4.65 (d, *J =* 2.0 Hz, 1H), 1.97–1.87 (m, 1H), 1.82–1.72 (m, 2H), 1.71–1.65 (m, 1H), 1.52 (tdd, *J =* 12.5, 5.8, 4.1 Hz, 1H), 1.27 (dd, *J =* 10.3, 1.5 Hz, 1H), 1.23 (s, 3H), 1.16 (s, 3H), 0.90 (s, 3H). ^13^C-NMR δ 166.16, 133.59, 132.33, 131.41, 131.10, 130.65, 126.56, 88.01, 48.49, 48.44, 41.46, 39.77, 29.71, 26.91, 25.85, 20.38, 19.56. HRMS (*m*/*z*) calcd. for C_17_H_22_ClO_2_^+^ (M + H)^+^ 293.13028, found 293.13025.

*(1S,2S,4R)-1,7,7-trimethylbicyclo[2.2.1]heptan-2-yl 4-chlorobenzoate* (**3o**): colorless oil, yield 75%. ^1^H- NMR δ 8.04–7.95 (m, 2H), 7.46–7.39 (m, 2H), 4.61 (d, *J* = 1.9 Hz, 1H), 1.94–1.86 (m, 1H), 1.78 (ddt, *J* = 9.0, 6.0, 2.9 Hz, 2H), 1.67 (dd, *J =* 10.4, 1.7 Hz, 1H), 1.54–1.47 (m, 1H), 1.28–1.19 (m, 2H), 1.18 (s, 3H), 1.10 (s, 3H), 0.83 (s, 3H). ^13^C-NMR δ 166.05, 139.22, 130.91, 129.15 (s), 128.74, 87.01, 48.63, 48.40), 41.46, 39.86, 29.75, 26.89, 25.91, 20.29, 19.49. HRMS (*m*/*z*) calcd. for C_17_H_22_ClO_2_^+^ (M + H)^+^ 293.13028, found 293.13031.

*(1S,2S,4R)-1,7,7-Trimethylbicyclo[2.2.1]heptan-2-yl 4-bromobenzoate* (**3p**): light yellow oil, yield 79%. ^1^H-NMR δ 7.96–7.88 (m, 2H), 7.63–7.54 (m, 2H), 4.61 (d, *J =* 1.9 Hz, 1H), 1.94–1.85 (m, 1H), 1.78 (ddt, *J* = 9.1, 5.8, 3.0 Hz, 2H), 1.66 (dd, *J* = 10.4, 1.7 Hz, 1H), 1.53–1.47 (m, 1H), 1.28–1.19 (m, 2H), 1.18 (s, 3H), 1.10 (s, 3H), 0.83 (s, 3H). ^13^C-NMR δ 166.18, 131.74, 131.06, 129.60, 127.86, 87.04, 48.63, 48.40, 41.46, 39.86, 29.75, 26.89, 25.90, 20.28, 19.49. HRMS (*m*/*z*) calcd. for C_17_H_22_BrO_2_^+^ (M + H)^+^ 337.07977, found 337.08023.

*(1S,2S,4R)-1,7,7-Trimethylbicyclo[2.2.1]heptan-2-yl 4-iodobenzoate* (**3q**): white solid, m.p. 112.9–114.5 °C, yield 85%. ^1^H-NMR δ 8.02 (dd, *J =* 7.9, 1.1 Hz, 1H), 7.83 (dd, *J =* 7.8, 1.7 Hz, 1H), 7.43 (td, *J =* 7.6, 1.2 Hz, 1H), 7.21–7.13 (m, 1H), 4.66 (d, *J =* 1.9 Hz, 1H), 1.95–1.86 (m, 1H), 1.82–1.73 (m, 2H), 1.72–1.66 (m, 1H), 1.52 (tdd, *J =* 12.5, 5.8, 4.1 Hz, 1H), 1.27 (dd, *J =* 10.3, 1.5 Hz, 1H), 1.24 (s, 3H), 1.18 (s, 3H), 0.91 (s, 3H). ^13^C-NMR δ 166.86, 141.35, 135.66, 132.41, 130.69, 127.88, 94.13, 87.81, 48.55, 48.42, 41.51, 39.85, 29.76, 26.88, 25.87, 20.47, 19.69. HRMS (*m*/*z*) calcd. for C_17_H_22_IO_2_^+^ (M + H)^+^ 385.06590, found 385.06583.

*(1S,2S,4R)-1,7,7-Trimethylbicyclo[2.2.1]heptan-2-yl 2-hydroxy-3,5-dinitrobenzoate* (**3r**): yellow powder, m.p. 108.1–109.2 °C, yield 79%. ^1^H-NMR δ 12.90 (s, 1H), 9.06 (d, *J* = 2.8 Hz, 1H), 8.98 (d, *J* = 2.9 Hz, 1H), 4.75 (d, *J =* 1.9 Hz, 1H), 1.87 (dd, *J =* 3.7, 2.2 Hz, 2H), 1.65–1.55 (m, 2H), 1.39–1.33 (m, 2H), 1.23 (s, 3H), 1.17 (s, 3H), 0.89 (s, 3H). ^13^C-NMR δ 168.35, 159.92, 129.55, 126.55, 90.53, 48.70, 48.23, 41.40, 40.09, 29.59, 26.81, 25.78, 20.32, 19.45. HRMS (*m*/*z*) calcd. for C_17_H_19_N_2_O_7_^−^ (M − H)^−^ 363.11977, found 363.11548.

*(1S,2S,4R)-1,7,7-Trimethylbicyclo[2.2.1]heptan-2-yl 4-cyanobenzoate* (**3s**): white solid, m.p. 115.2–117.4 °C, yield 80%. ^1^H-NMR δ 8.19–8.15 (m, 2H), 7.80–7.75 (m, 2H), 4.66 (d, *J* = 1.9 Hz, 1H), 1.95–1.86 (m, 1H), 1.84–1.75 (m, 2H), 1.72–1.66 (m, 1H), 1.55 (tdd, *J* = 12.6, 5.7, 4.1 Hz, 1H), 1.29 (dd, *J* = 10.5, 1.6 Hz, 1H), 1.20 (s, 3H), 1.13 (s, 3H), 0.85 (s, 3H). ^13^C-NMR δ 165.21, 134.47, 132.27, 130.00, 118.03, 116.25, 87.70, 48.63, 48.35, 41.42, 39.89, 39.88, 29.73, 26.86, 25.87, 20.27, 19.47. HRMS (*m*/*z*) calcd. for C_18_H_22_NO_2_^+^ (M + H)^+^ 284.16451, found 284.16534.

*(1S,2S,4R)-1,7,7-Trimethylbicyclo[2.2.1]heptan-2-yl 2-(naphthalen-1-yl)acetate* (**3t**): colorless oil, yield 94%. ^1^H-NMR δ 8.08–7.99 (m, 1H), 7.84 (t, *J* = 8.8 Hz, 1H), 7.81–7.74 (m, 1H), 7.56–7.45 (m, 2H), 7.44–7.39 (m, 2H), 4.33 (s, 1H), 4.10 (d, *J* = 1.5 Hz, 2H), 1.63 (d, *J* = 1.3 Hz, 1H), 1.59–1.40 (m, 4H), 1.35 (tt, *J =* 10.9, 5.8 Hz, 1H), 1.10 (d, *J =* 10.2 Hz, 1H), 1.02 (d, *J =* 2.4 Hz, 3H), 0.89 (d, *J =* 2.3 Hz, 3H), 0.57 (d, *J* = 2.2 Hz, 3H). ^13^C-NMR δ 166.98, 143.41, 129.58, 129.09, 128.01, 86.45, 48.64, 48.45, 41.48, 39.85, 29.77, 26.91, 25.94, 21.66, 20.31, 19.51. HRMS (*m*/*z*) calcd. for C_22_H_27_O_2_^+^ (M + H)^+^ 323.20056, found 323.20062.

### 3.3. Insecticidal Activity

All the insecticidal bioassays were performed using representative test organisms prepared in the laboratory. The bioassay was performed at 25 ± 1 °C. All the compounds were dissolved in DMF and diluted with 0.05% Triton X-100 to obtain a series of concentrations. The insecticidal activities of the compounds against *H. armigera* (Hübner), *T. truncatus* and *T. turkestani* Ugarov et Nikolski were tested according to previously reported procedures [18]. Each administration was repeated three times. Chlorantraniliprole, biflenazate, pyridaben and hexythiazox purchased from J&K Scientific Ltd., were used as a control and used in the same way.

### 3.4. Enzyme Activity

The enzyme activity was measured according to the assay kit instructions provided by the manufacturer (Nanjing Jiancheng Bioengineering Institute). Sample preparation and purification were performed as follows: 0.1 g of *H. armigera* tissues were accurately weighed, a 1 mL extracted solution and homogenate added under an ice bath, and the supernatant placed on the ice after centrifugation (chitinase: 12,000 rmp, 4 °C, centrifugation for 20 min; carboxylic acid esterase: 12,000 rmp, 4 °C, centrifugation for 30 min; glutathione *S*-transferase: 10,000 rmp, 4 °C centrifugation for 10 min; polyphenol oxidase: 8000 rmp, room temperature centrifugation 10 min). The sample pretreatment of acid phosphatase and alkaline phosphatase was as follows: 0.1 g of *H. armigera* tissues were accurately weighed, 0.9 mL of physiological saline and homogenate was added under an ice bath, then the mixture was centrifugation at 2500 rpm for 10 min, and the supernatant was determined.

### 3.5. Antifungal Activity

The antifungal activities of the synthesized compounds were performed according to previously reported procedures [19]. The fungicidal activities of the target compounds against *C. lagenarium* (Pass.) Ell. et Halst, *R. solani*, *F. fulva* (Cooke) Cif, *P. grisea* (Cooke) Sacc, *A. alternata* Japanese pear pathotype, sunflower sclerotinia rot and rape sclerotinia rot were evaluated using a mycelium growth rate test. Azoxystrobin, kresoxim-methyl, and trifloxystrobin purchased from J&K Scientific Ltd., were used as controls, and were utilized in the same way. The relative inhibition ratio (%) was calculated:

$$\mathrm{The}\;\mathrm{relative}\;\mathrm{inhibition}\;\mathrm{ratio}\;\left(\%\right)=\frac{\mathrm{colony}\;\mathrm{diameter}\;\mathrm{of}\;\mathrm{control}-\;\mathrm{colony}\;\mathrm{diameter}\;\mathrm{of}\;\mathrm{treated}}{\mathrm{colony}\;\mathrm{diameter}\;\mathrm{of}\;\mathrm{control}\;\mathrm{mycelial}\;\mathrm{disk}\;\mathrm{diameter}}\times 100\%$$

### 3.6. Quantitative Structure–Activity Relationship (QSAR) Analyses

Three-dimensional QSAR analyses were performed to predict the favorable and unfavorable moieties for improved bioactivity using the CoMFA models in the SYBYL7.3 software. CoMFA models were generated using the Sybyl 7.3 package (Certara, Princeton, NJ, USA) on a Linux system. In total, 20 compounds obtained from synthesis were used to create a data set in which the bioactivity of all compounds were determined against *T. turkestani* Ugarov et Nikolski. Three-dimensional molecular structures were built using the SKETCH module in Sybyl 7.3, while structural energy minimization was performed with the Tripos force field until a gradient convergence of 0.05 kcal/(mol A) was achieved. Gasteiger–Hückel charges were calculated and used to construct the CoMFA models [20].

## 4. Conclusions

In summary, we have designed and synthesized a novel series of the title compounds based on the active structure of tschimganin, and assayed their in vitro insecticidal, acaricidal and fungicidal activity against seven fungi. Compounds **3d** and **3g**, with electron donating groups, showed better inhibitory activity and growth inhibition activity to *H. armigera* (Hübner). Morphological and enzymatic assays demonstrated that the compounds **3d** and **3g** could effectively inhibit the growth and development of cotton bollworm. The antifungal evaluation showed that the properties and positions of these substituents on the benzene ring have an important influence on the acaricidal activity of tschimganin analogs. The 3D-QSAR model for the compounds against *T. turkestani* Ugarov et Nikolski indicated that the electronegativity was beneficial for acaricidal activity. Notably, compound **3r** having a 2-hydroxy-3,5-dinitrophenyl moiety displayed a fungicidal spectrum as broad as that of azoxystrobin against these phytopathogens. All results suggested these compounds have potential to be used as bifunctional agrochemicals. Further studies on structural optimization are in progress at our laboratory.

## Figures and Tables

**Table 1:** In vitro inhibitory activity against the cotton bollworm and the two cotton spider mite species at 50 μg/mL ^a,^^b^.

Compound	*H. armigera* (Hübner)		*T. truncatus*		*T. turkestani*	
	Mortality Rate % (48 h)	Growth and Development	Mortality Rate % (24 h)	Mortality Rate % (48 h)	Mortality Rate % (24 h)	Mortality Rate % (48 h)
**3** **a**	0	ND	15.56	63.33	10.41	40.62
**3** **b**	3	dysplasia	13.33	48.89	8.93	50.00
**3** **c**	10	dysplasia	27.78	45.56	22.29	46.87
**3** **d**	37	dysplasia	55.56	83.33	26.75	35.94
**3** **e**	30	dysplasia	30.00	40.00	25.27	71.87
**3** **f**	23	dysplasia	38.89	52.22	47.57	70.31
**3** **g**	60	dysplasia	64.44	93.33	28.24	48.44
**3** **h**	13	dysplasia	17.89	35.67	60.95	79.69
**3** **i**	17	dysplasia	84.44	93.33	53.51	64.06
**3** **j**	7	dysplasia	33.33	37.78	74.33	82.81
**3** **k**	0	ND	18.89	33.33	11.89	37.50
**3** **l**	0	ND	15.56	32.22	16.36	62.50
**3** **m**	0	ND	65.56	82.22	13.37	31.25
**3** **n**	10	dysplasia	15.56	30.00	13.37	62.50
**3** **o**	10	dysplasia	12.22	16.67	5.94	18.75
**3** **p**	7	dysplasia	11.11	17.78	25.27	21.87
**3** **q**	10	dysplasia	13.23	15.36	41.62	59.37
**3** **r**	0	ND	15.56	51.11	-	-
**3** **s**	7	dysplasia	12.22	22.22	35.67	50.00
**3** **t**	0	ND	17.78	41.11	5.95	0.00
chlorantraniliprole	100	ND	-	-	-	-
biflenazate	-	-	85.56	95.56	61.11	65.62
pyridaben	-	-	61.11	71.21	97.78	98.44
hexythiazox	-	-	83.33	83.33	67.19	71.11

^a^ “-” means activity is not tested; ^b^ “ND” means no dysplasia. The data in bold are used to emphasize that these compounds showed good activity.

**Table 2:** In vitro inhibitory activity against plant pathogens at 50 μg/mL ^a^.

Compound	Inhibition Ratio (%)						
	CL	RS	FF	PG	AK	SSR	RSR
**3** **a**	17.89	20.87	31.74	21.09	37.32	32.32	**56.75**
**3** **b**	0.00	−4.81	4.63	32.43	11.97	**55.49**	**67.86**
**3** **c**	0.00	−9.40	8.37	23.36	37.32	39.23	36.31
**3** **d**	0.00	**40.04**	29.64	37.19	40.85	49.39	22.62
**3** **e**	0.00	10.02	**50.44**	**60.09**	**57.04**	**49.59**	29.56
**3** **f**	1.42	−3.76	15.38	19.73	25.35	1.22	11.71
**3** **g**	0.00	−2.72	3.46	31.97	22.54	42.89	4.96
**3** **h**	0.00	6.89	14.91	24.49	37.32	36.18	41.87
**3** **i**	0.00	−8.98	17.02	27.44	41.55	26.63	**73.81**
**3** **j**	0.00	−8.98	8.60	29.71	31.69	7.72	**80.95**
**3** **k**	19.51	4.17	22.86	24.94	40.85	13.21	18.85
**3** **l**	15.04	−5.22	31.98	32.43	33.10	46.34	16.27
**3** **m**	28.46	−6.06	26.60	35.83	41.55	**57.93**	**50.60**
**3** **n**	0.00	17.32	32.21	36.51	11.97	22.97	16.27
**3** **o**	0.00	−10.03	15.61	24.94	26.76	17.80	8.13
**3** **p**	0.00	−5.43	13.51	30.61	38.03	2.85	14.29
**3** **q**	0.00	7.30	4.63	31.29	**63.38**	**55.49**	**78.57**
**3** **r**	**73.17**	**57.41**	**50.44**	**56.43**	**60.14**	**65.04**	**84.33**
**3** **s**	7.11	5.22	22.86	34.92	42.25	31.10	84.13
**3** **t**	6.30	−2.72	17.02	33.33	7.04	3.46	4.96
**azoxystrobin**	66.54	18.37	55.61	73.92	61.97	89.63	90.48
**kresoxim-methyl**	0.00	1.25	25.20	21.54	19.25	2.24	75.00
**trifloxystrobin**	46.54	28.60	52.55	71.28	65.39	86.79	17.26

^a^ CL: *C. lagenarium* (Pass.) Ell. et Halst; RS: *R. solani*; FF: *F. fulva* (Cooke) Cif.; PG: *P. grisea* (Cooke) Sacc.; AK: *A. alternata* Japanese pear pathotype; SSR: Sunflower sclerotinia rot; RSR: Rape sclerotinia rot. The data in bold are used to emphasize that these compounds showed good activity.

## Supplementary Materials

Supplementary data (i.e., the ^1^H-, ^13^C-NMR and HRMS spectra of all compounds mentioned in this article) are available online.
