# Supplementary material for: Synthesis, Insecticidal, Fungicidal Activities and Structure–Activity Relationships of Tschimganin Analogs
Source: Molecules. 2018 Jun 18;23(6):1473. doi: 10.3390/molecules23061473 (PMC6099738; doi:10.3390/molecules23061473)
Supplement: Supplementary file 1 [file molecules-23-01473-s001.zip › molecules-310568-supplementary.pdf]

Supplementary Material

Synthesis, Insecticidal, Fungicidal Activity and Structure−Activity Relationships of Tschimganin Analogs

Yueting Zhou[1], Chunjuan Wang[1], Fang Xin[1], Xiaoqiang Han[1*], Jie Zhang[2*], Ke Sun[3]

1. Key Laboratory at Universities of Xinjiang Uygur Autonomous Region for Oasis Agricultural Pest Management and Plant Protection Resource Utilization / College of Agricultural, Shihezi University, Shihezi 832002, China
2. Key Laboratory for Green Processing of Chemical Engineering of Xinjiang Production and Construction Group / School of Chemistry and Chemical Engineering, Shihezi 832003, Xinjiang, China
3. State Key Laboratory of the Discovery and Development of Novel Pesticide / Shenyang Sinochem Agrochemicals R&D Co. Ltd., No.8-1 Shenliao Dong Road, Tiexi District, Shenyang 110021, Liaoning, China

* Corresponding author. Tel. +86-10-2058060

E-mail: hanshz@shzu.edu.cn and zhangjie-xj@163.com

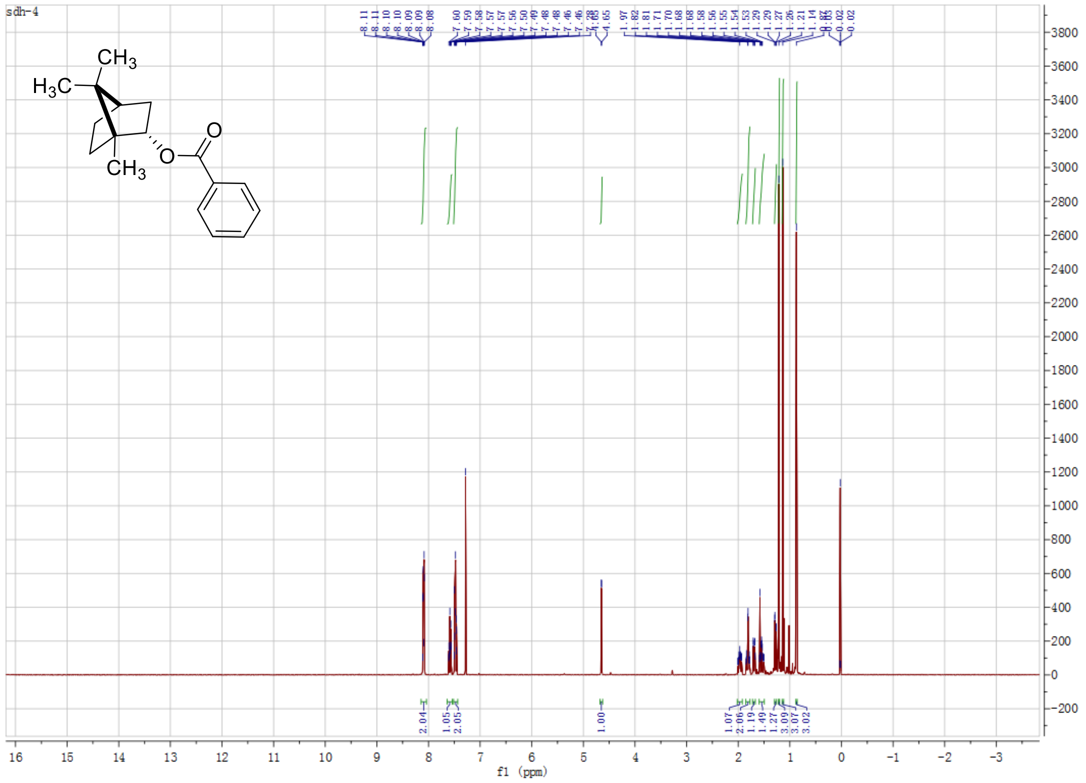

Figure 1 ^{1}H-NMR of **3**a

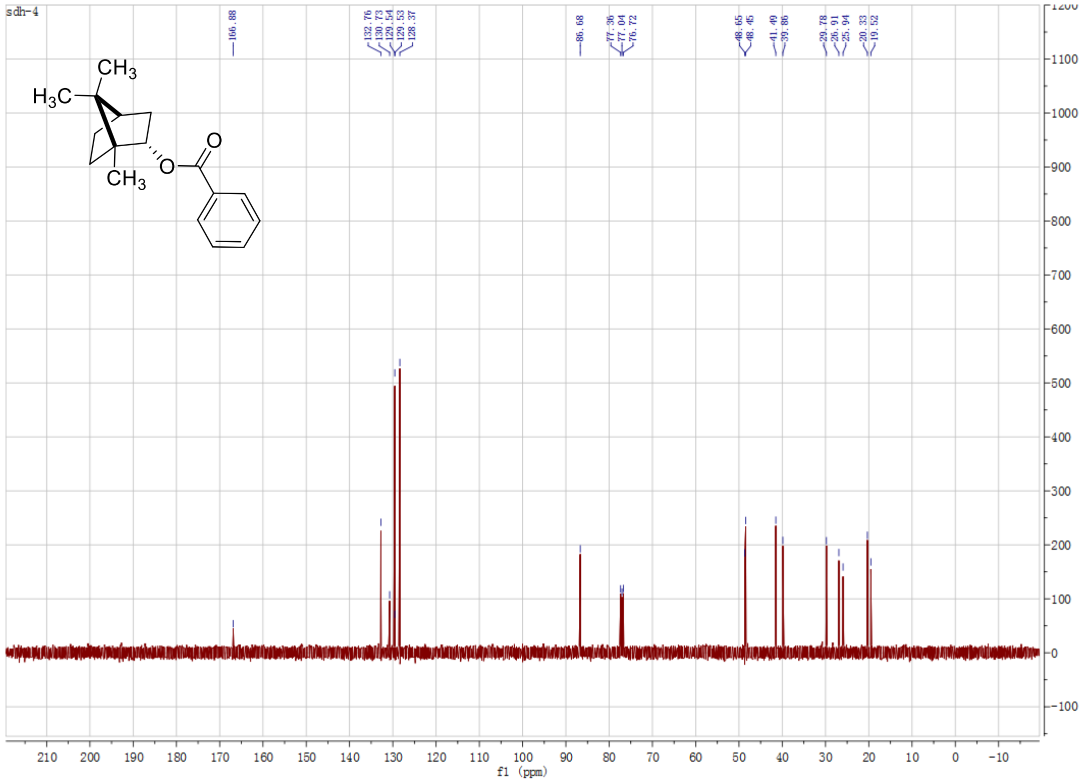

Figure 2 ^{13}C-NMR of **3**a

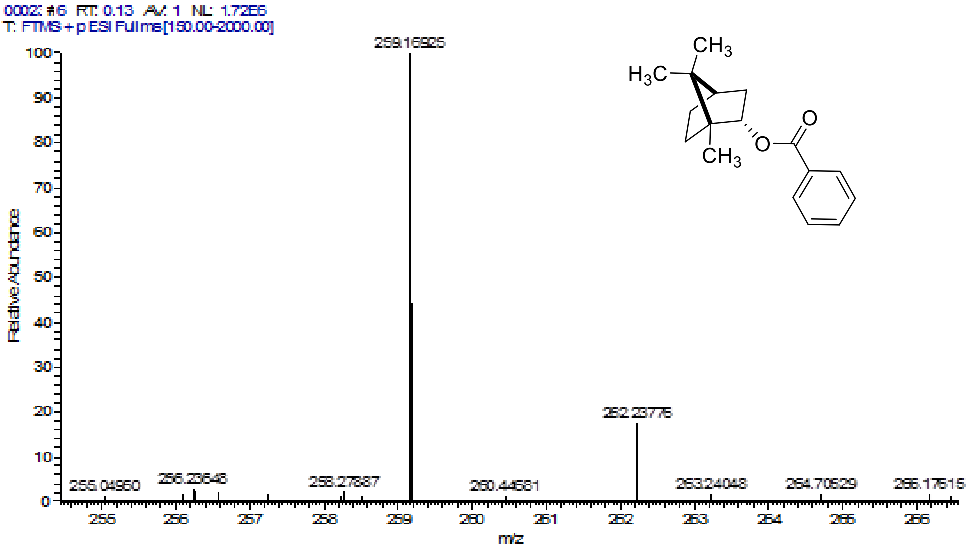

Figure 3 HRMS of **3**a

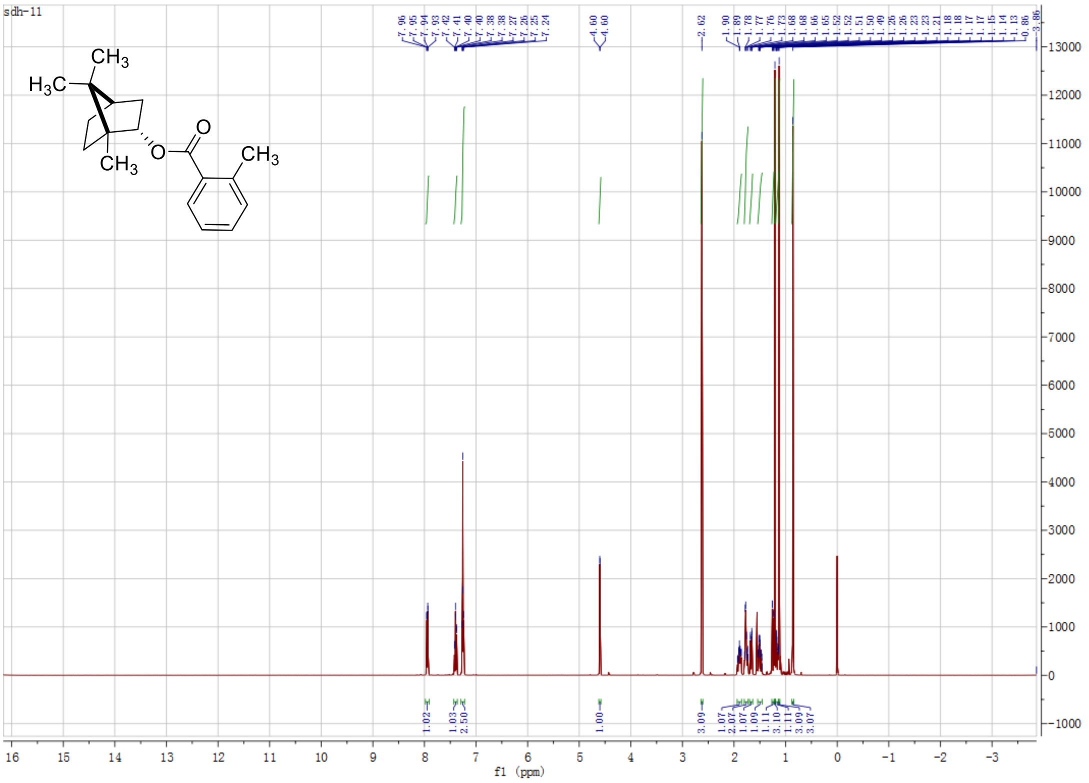

Figure 4 ^{1}H-NMR of **3**b

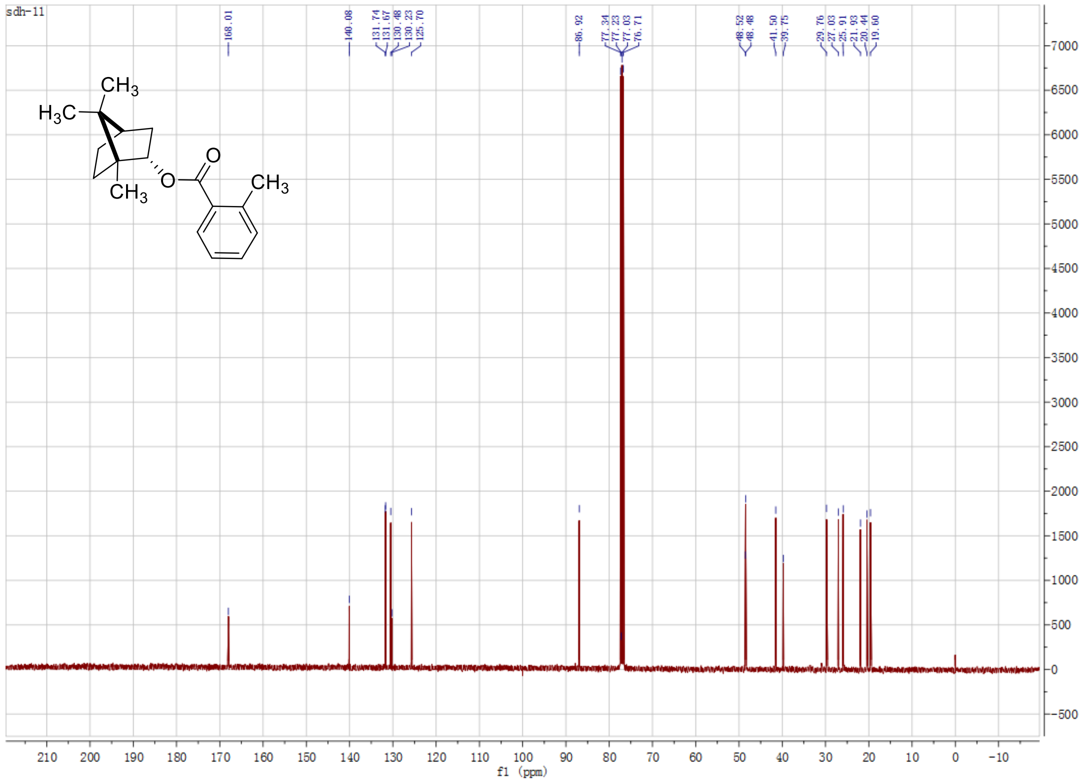

Figure 5 ^{13}C-NMR of **3**b

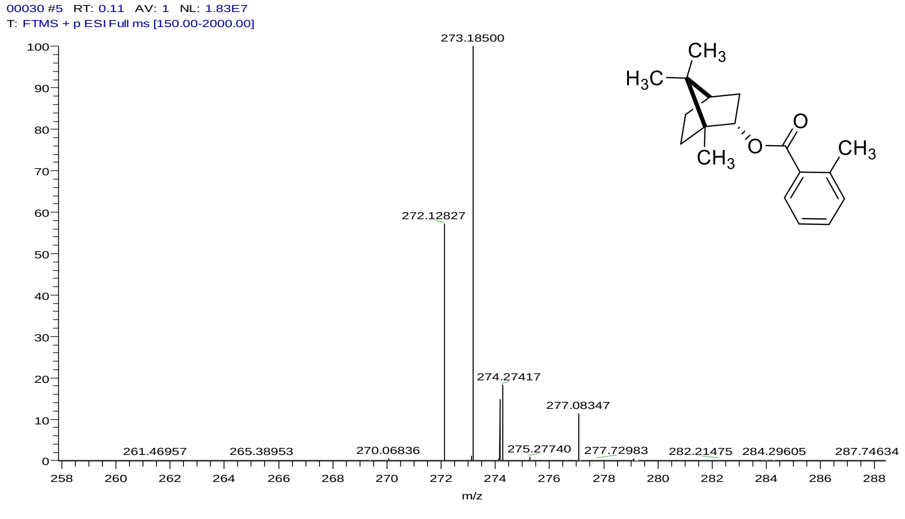

Figure 6 HRMS of **3**b

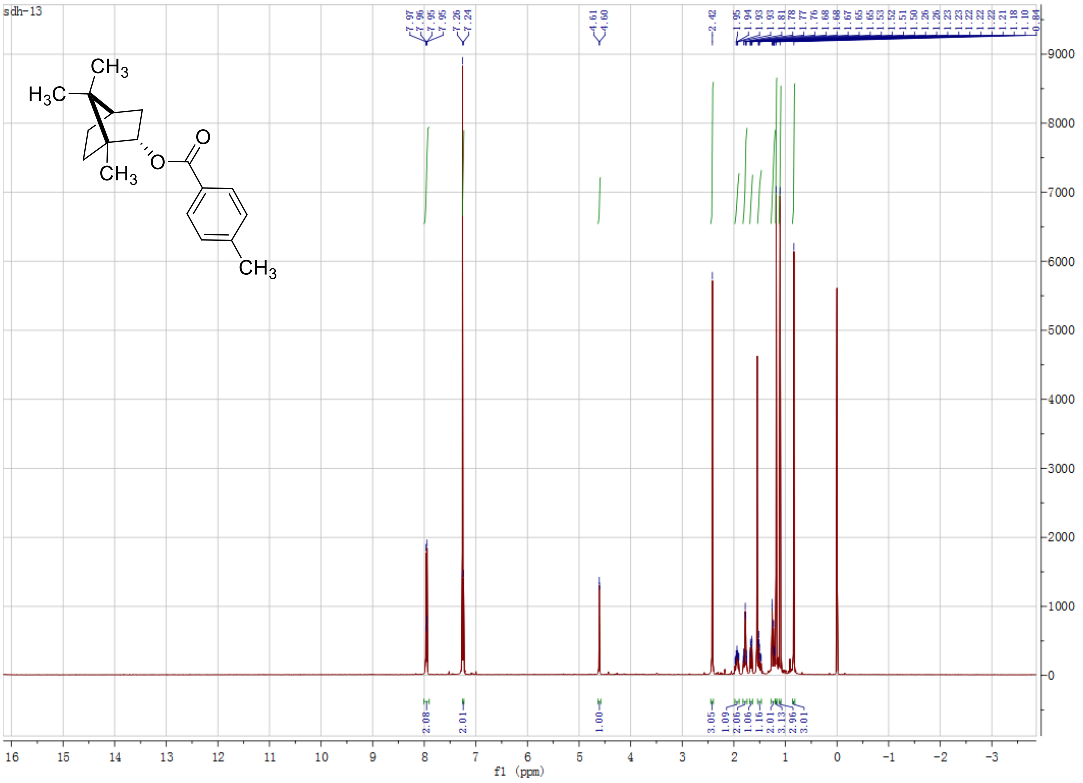

Figure 7 ^{1}H-NMR of **3**c

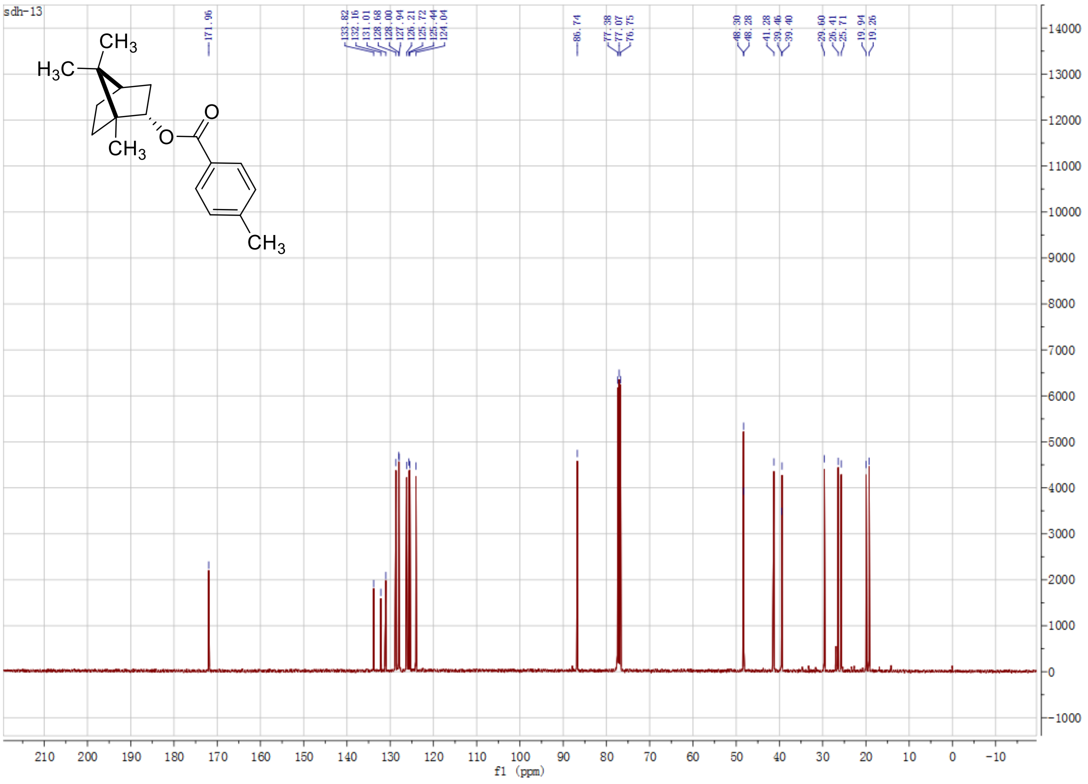

Figure 3 ^{13}C-NMR of **3**c

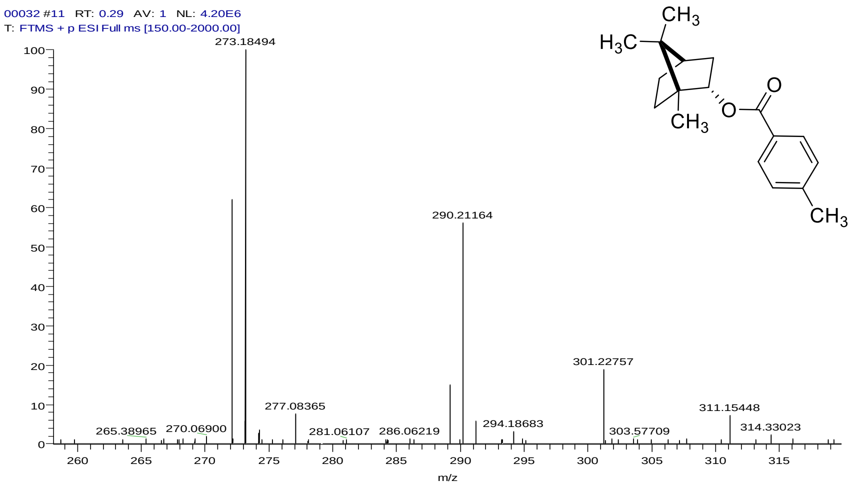

Figure 9 HRMS of **3**c

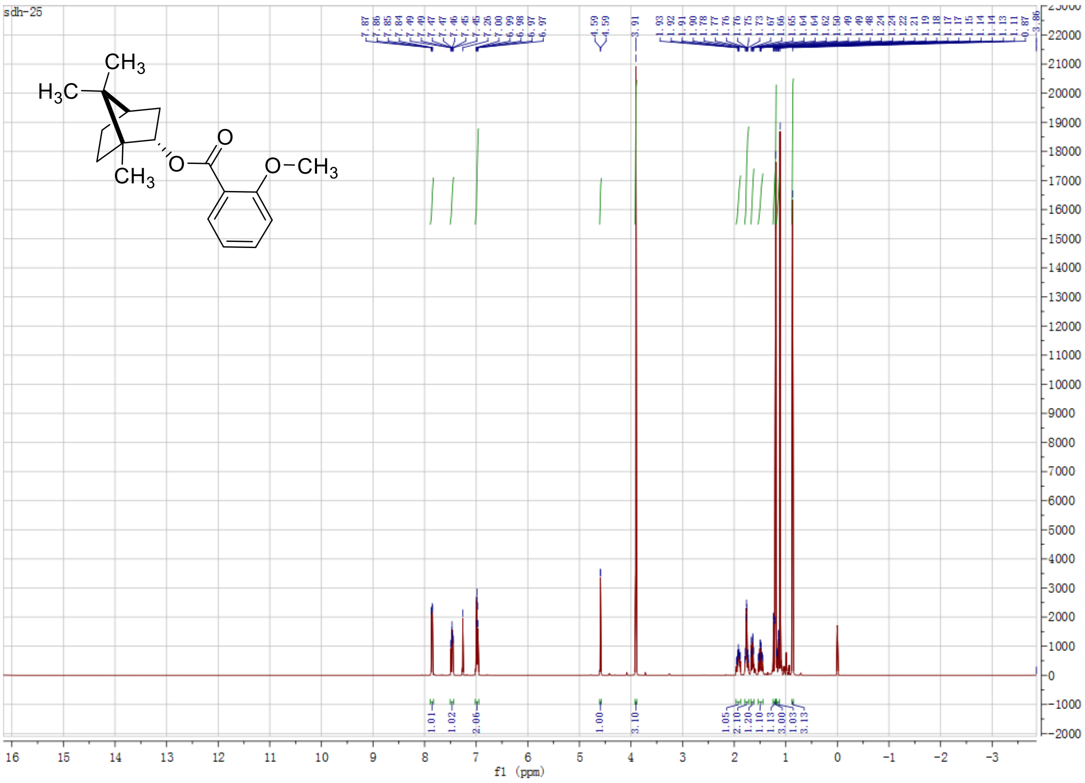

Figure 10 ^{1}H-NMR of **3**d

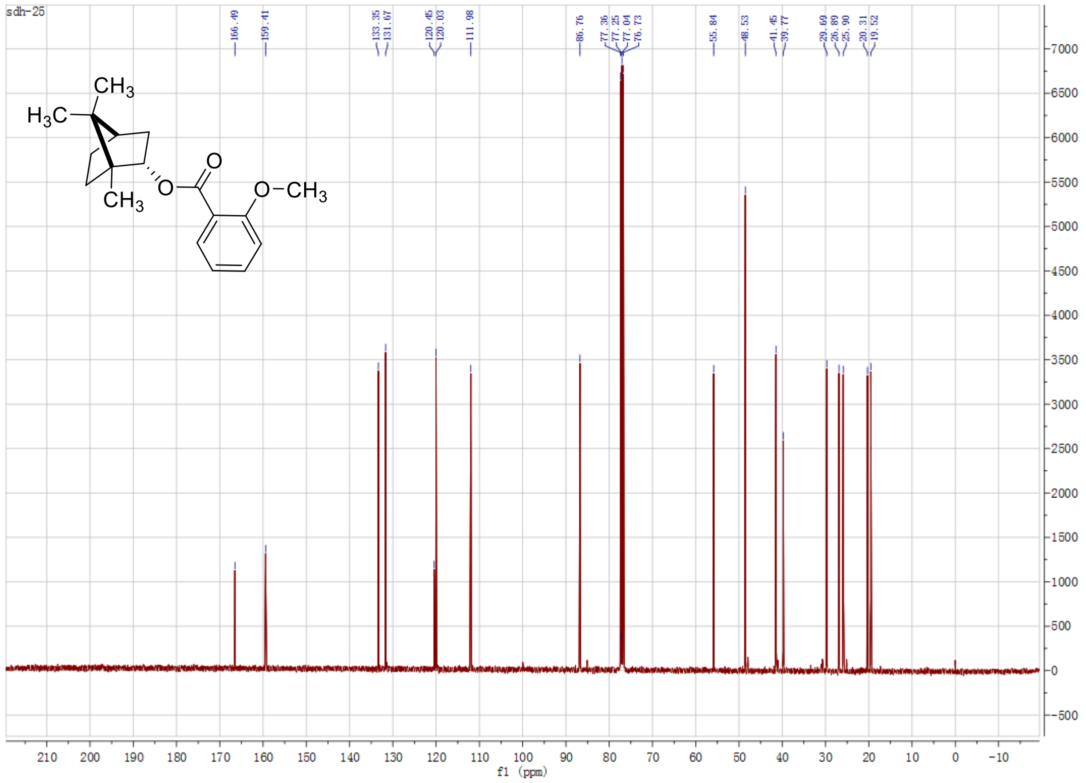

Figure 11 ^{13}C-NMR of **3**d

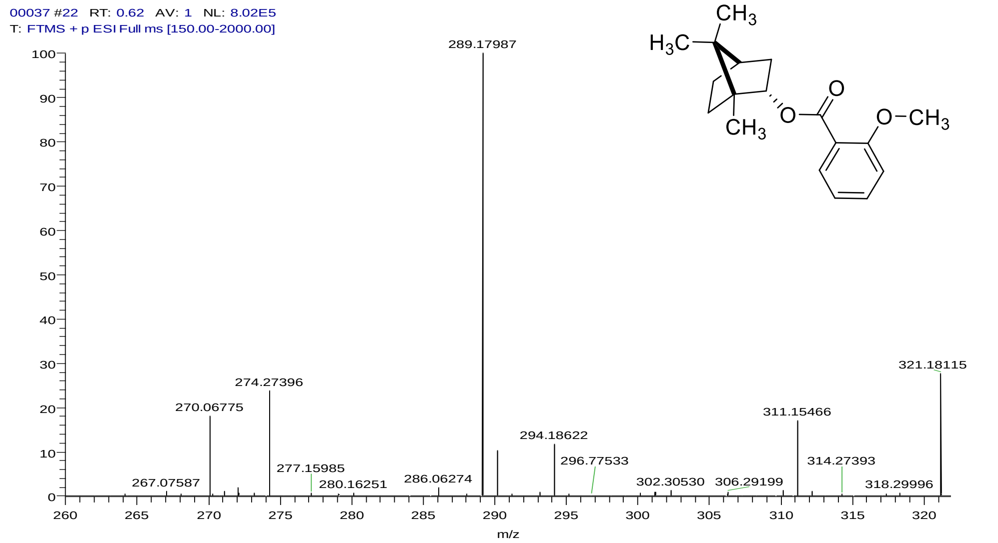

Figure 12 HRMS of **3**d

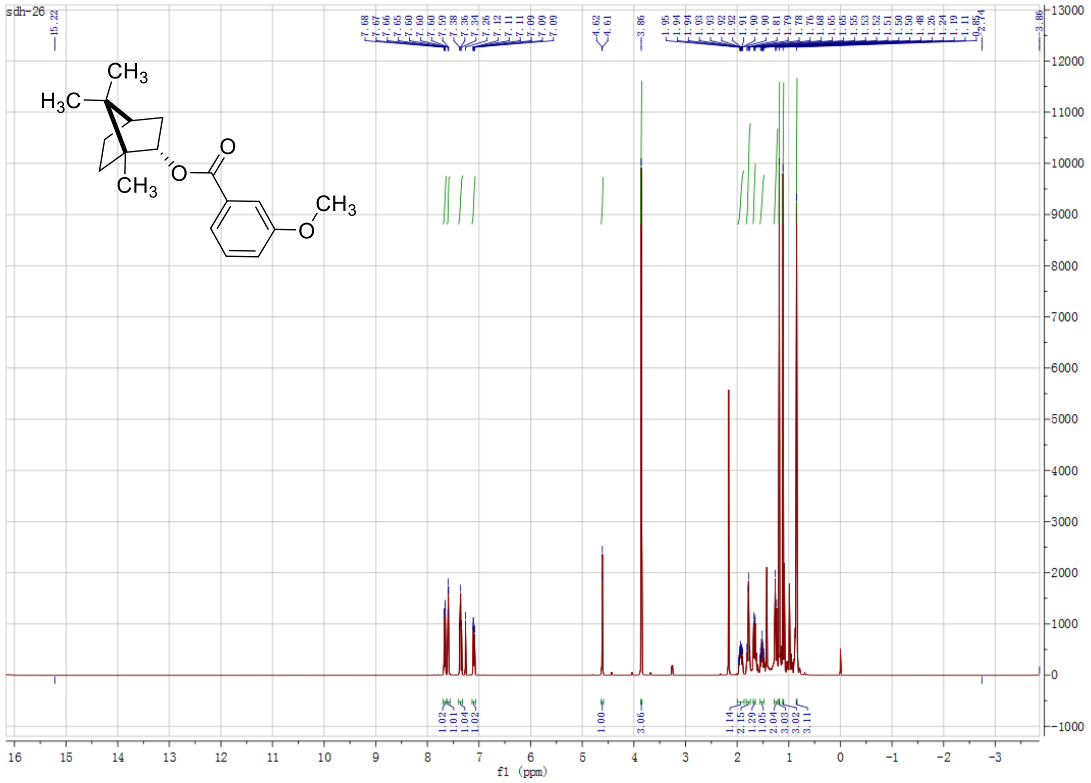

Figure 13 ^{1}H-NMR of **3**e

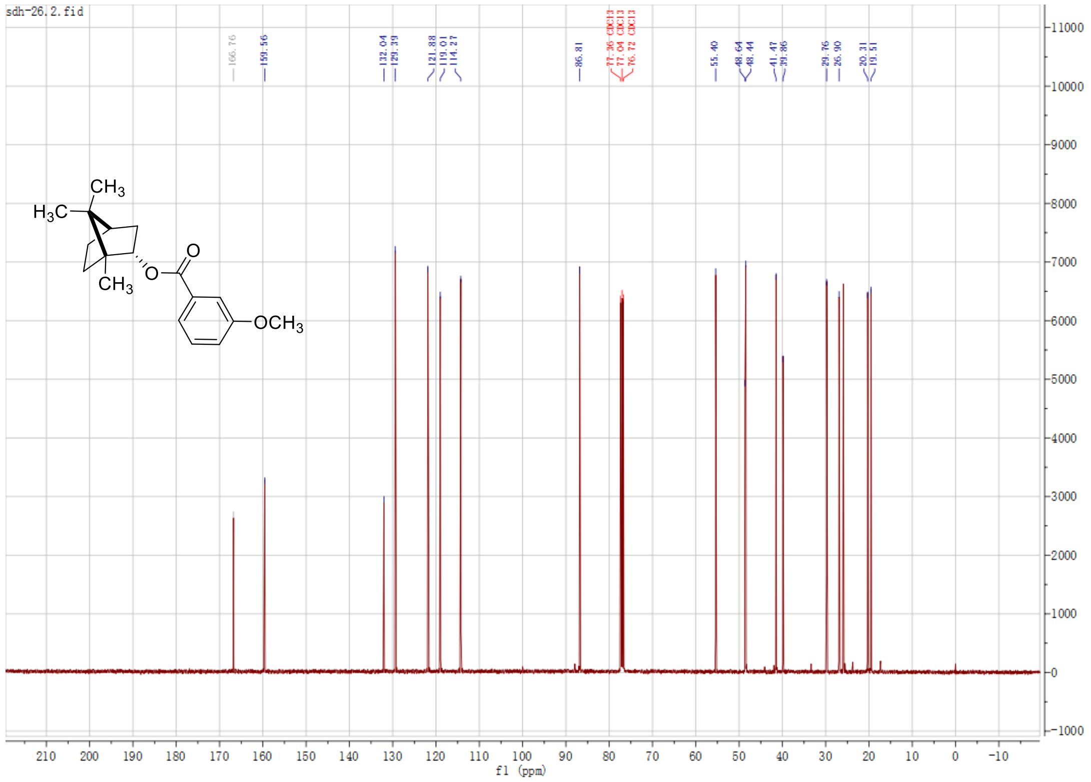

Figure 14 ^{13}C-NMR of **3**e

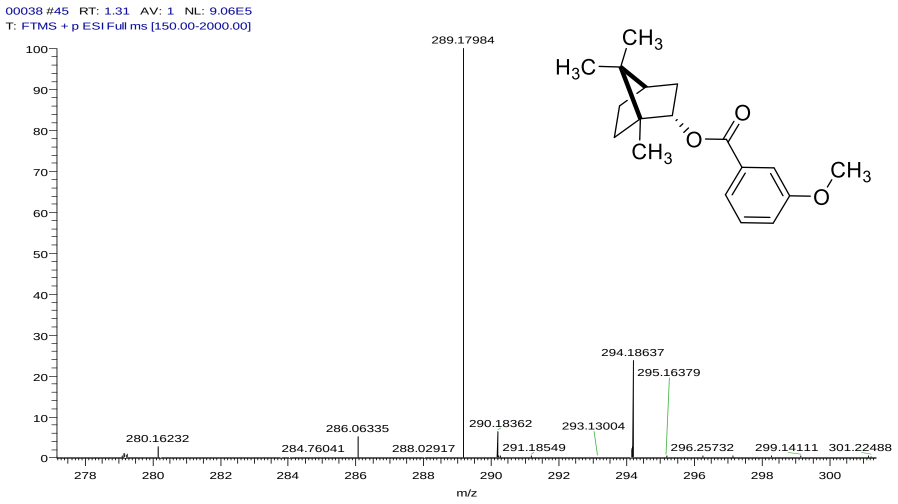

Figure 15 HRMS of **3**e

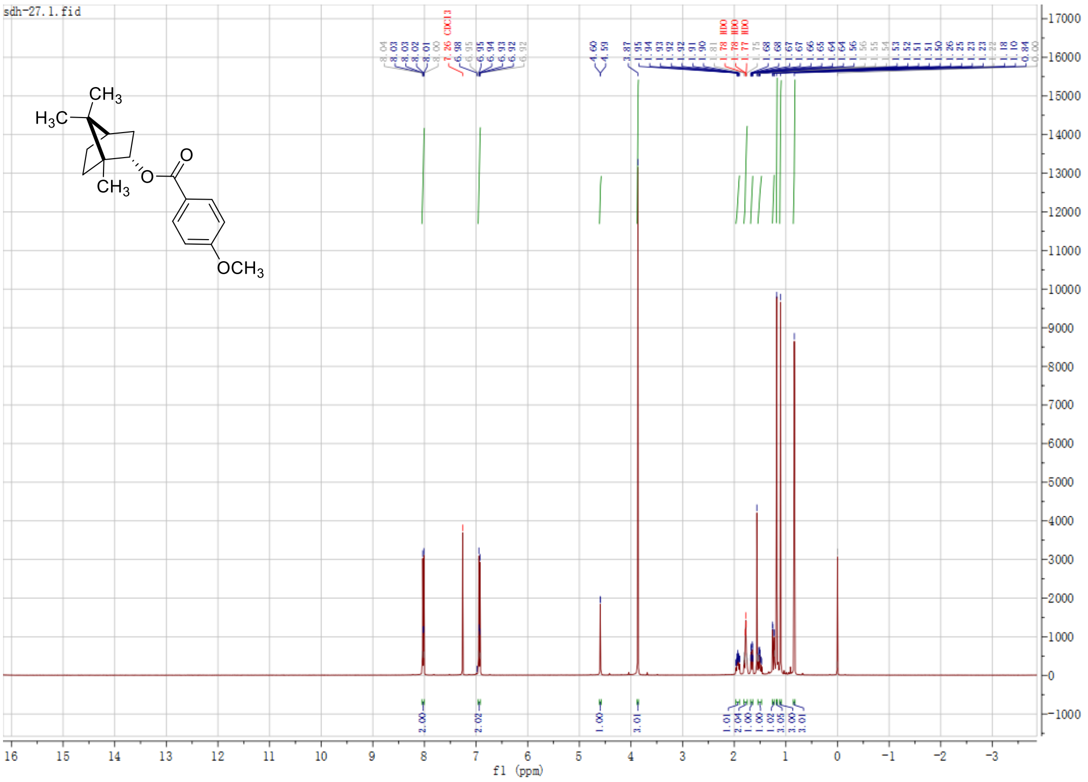

Figure 16 ^{1}H-NMR of **3**f

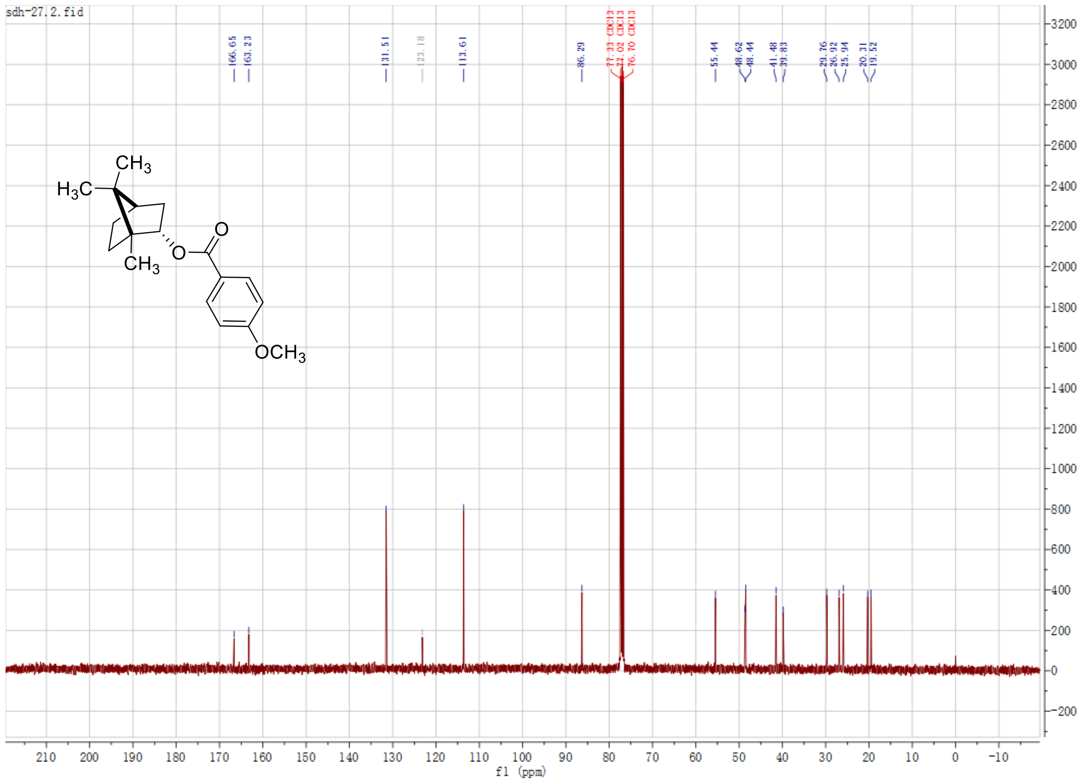

Figure 17 ^{13}C-NMR of 3f

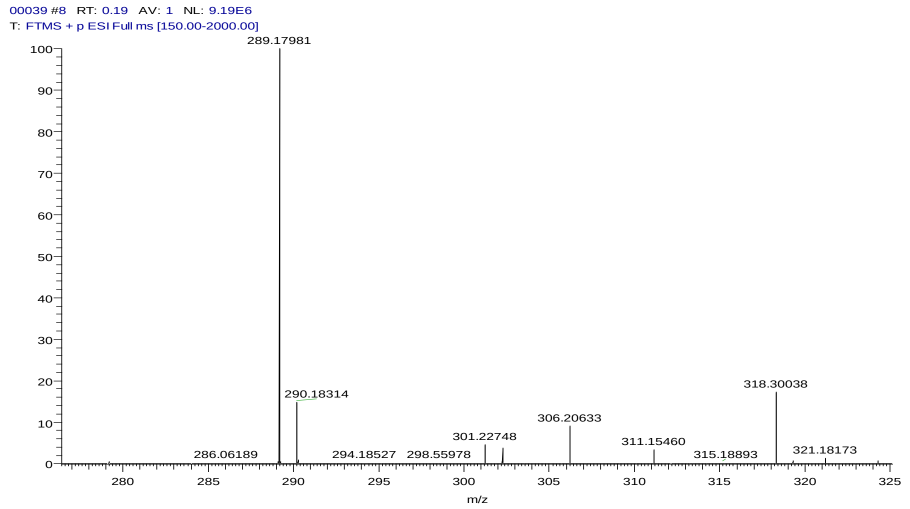

Figure 13 HRMS of **3**f

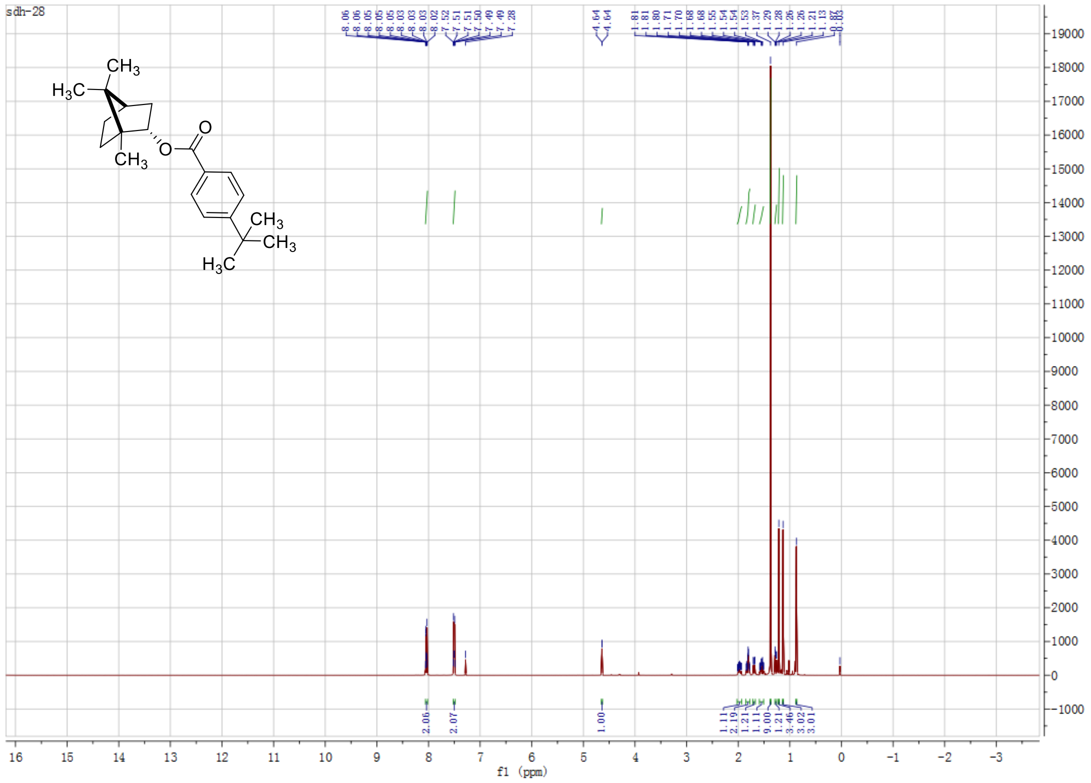

Figure 19 ^{1}H-NMR of **3**g

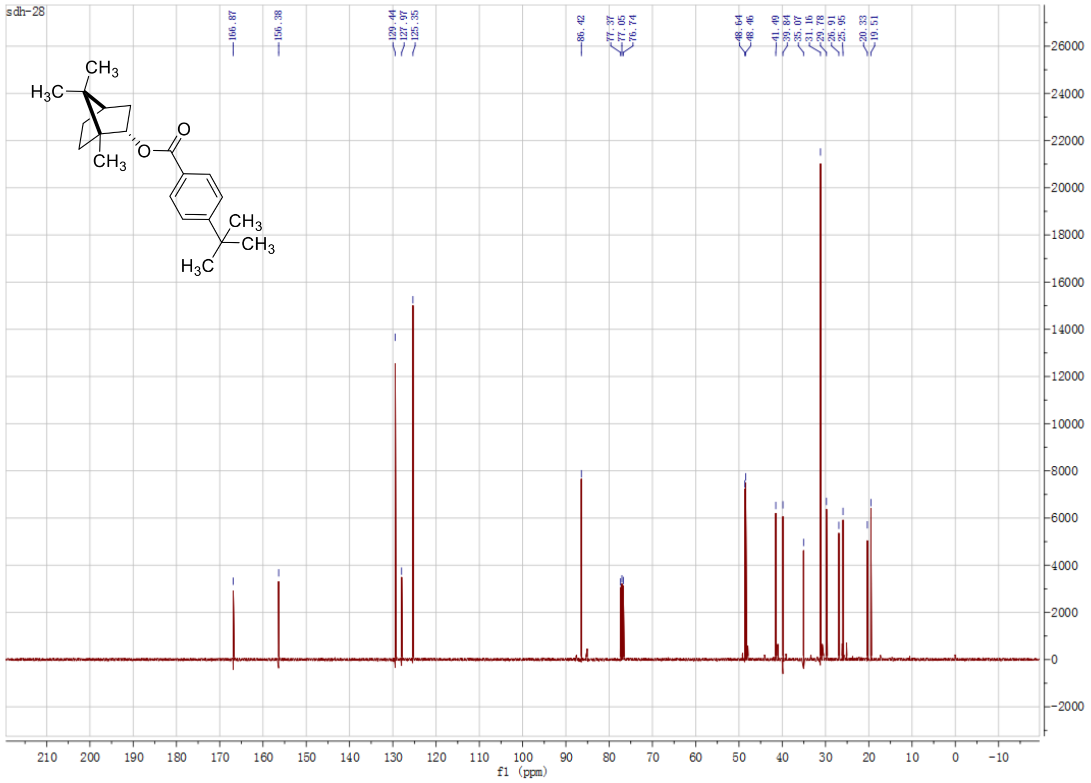

Figure 20 ^{13}C-NMR of **3**g

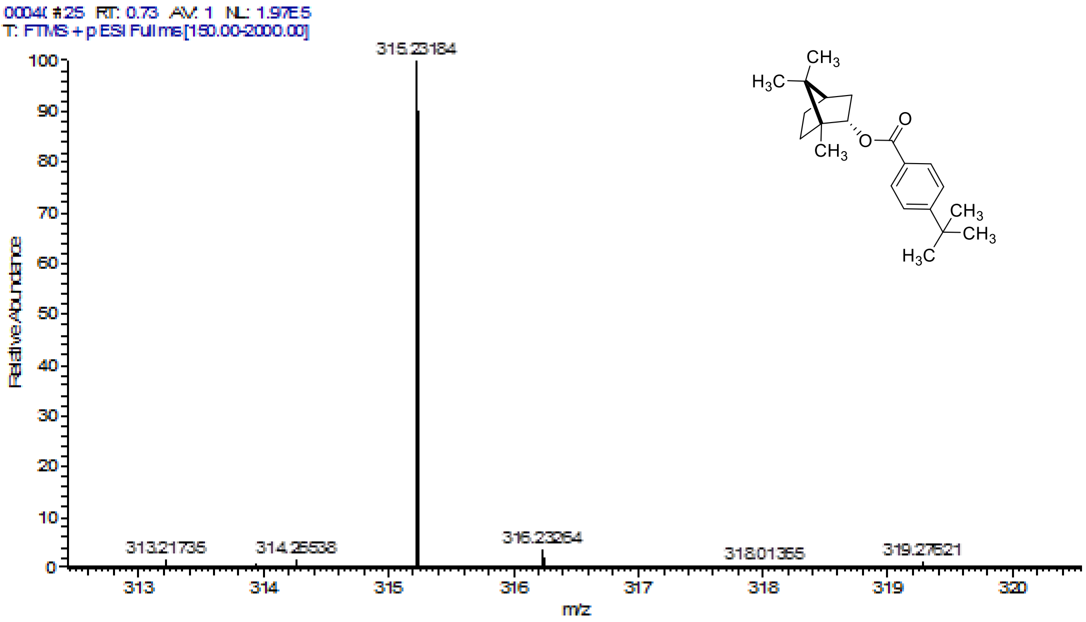

Figure 21 HRMS of **3**g

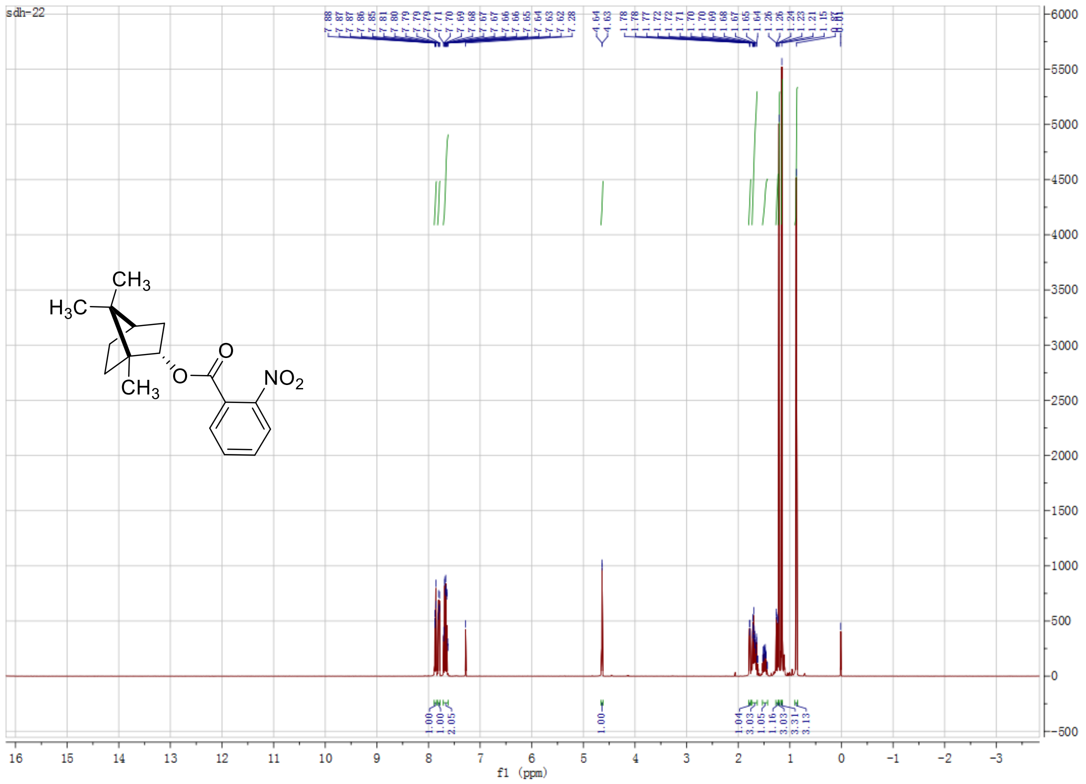

Figure 22 ^{1}H-NMR of **3**h

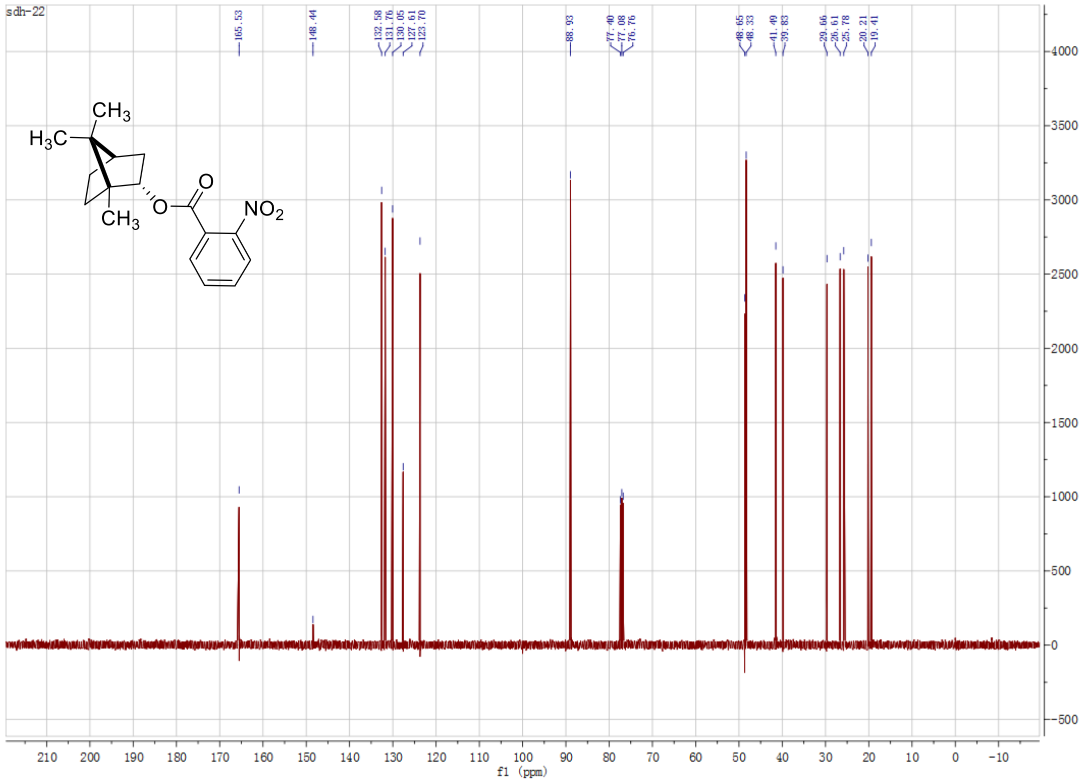

Figure 23 ^{13}C-NMR of **3**h

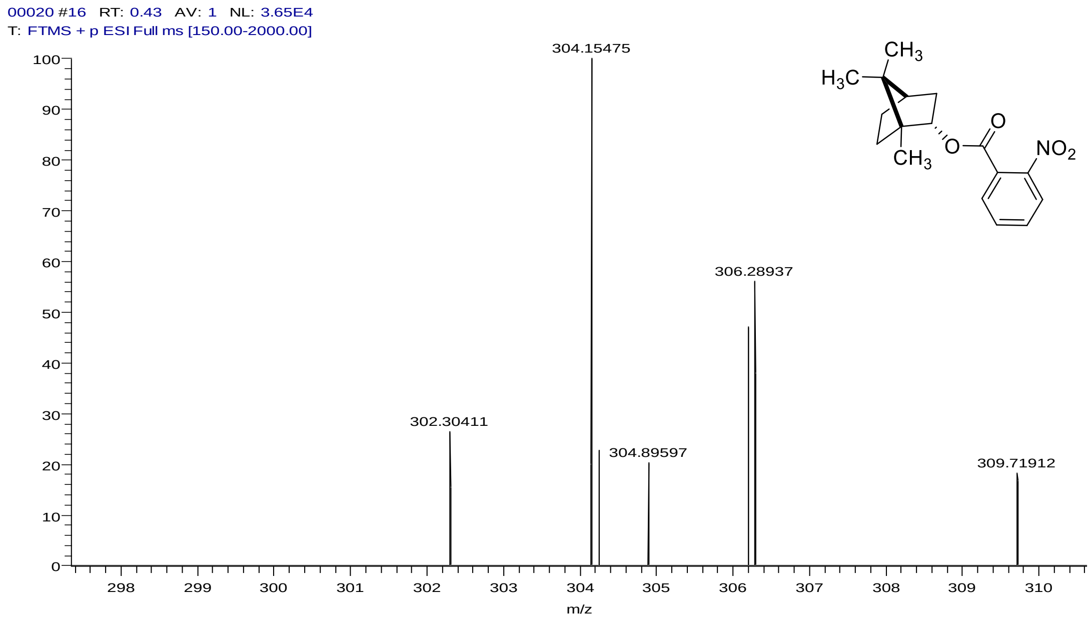

Figure 24 HRMS of **3**h

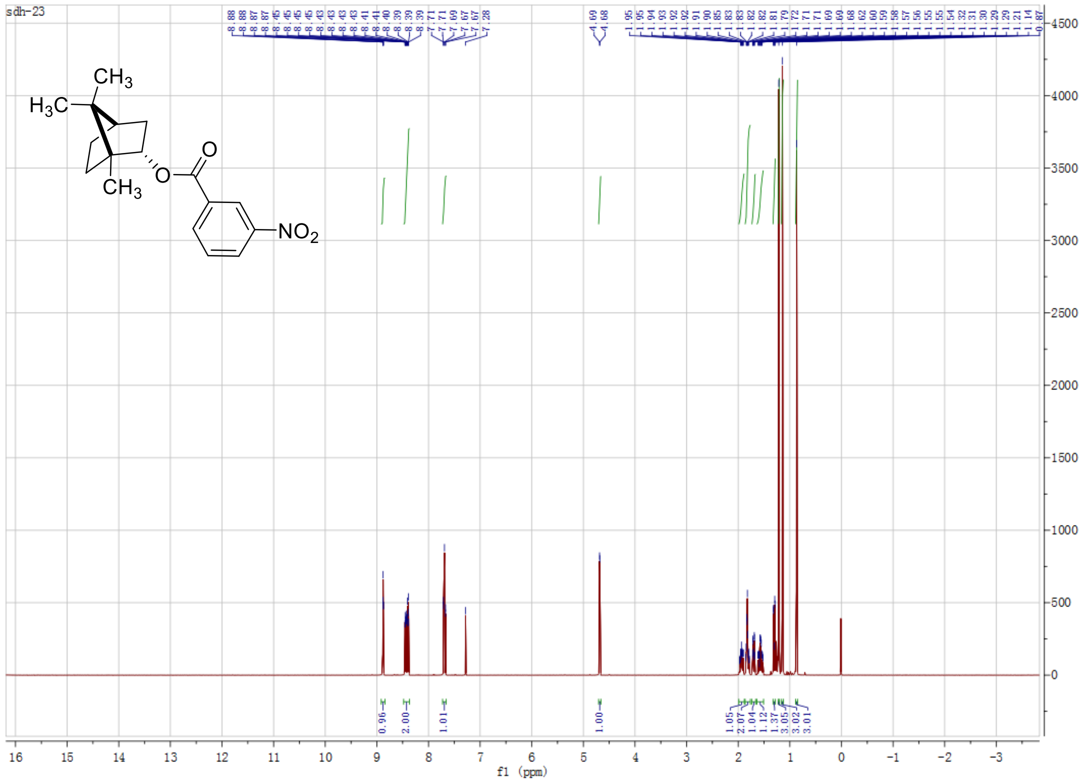

Figure 25 ^{1}H-NMR of **3**i

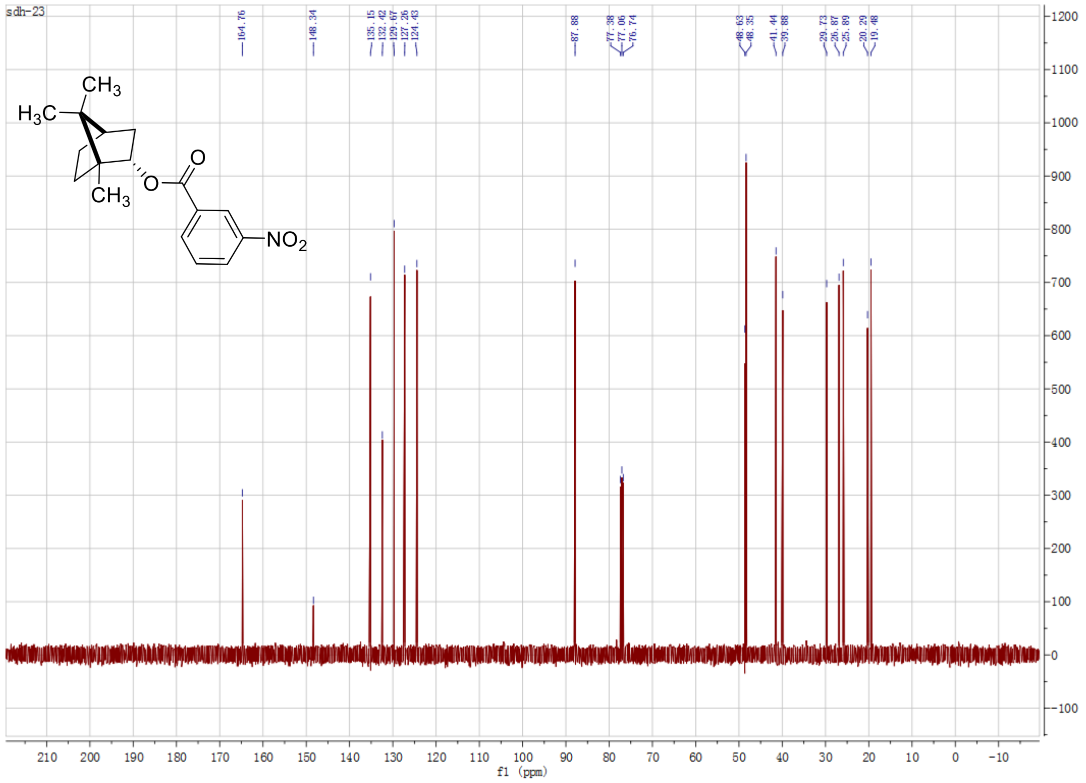

Figure 26 ^{13}C-NMR of **3**i

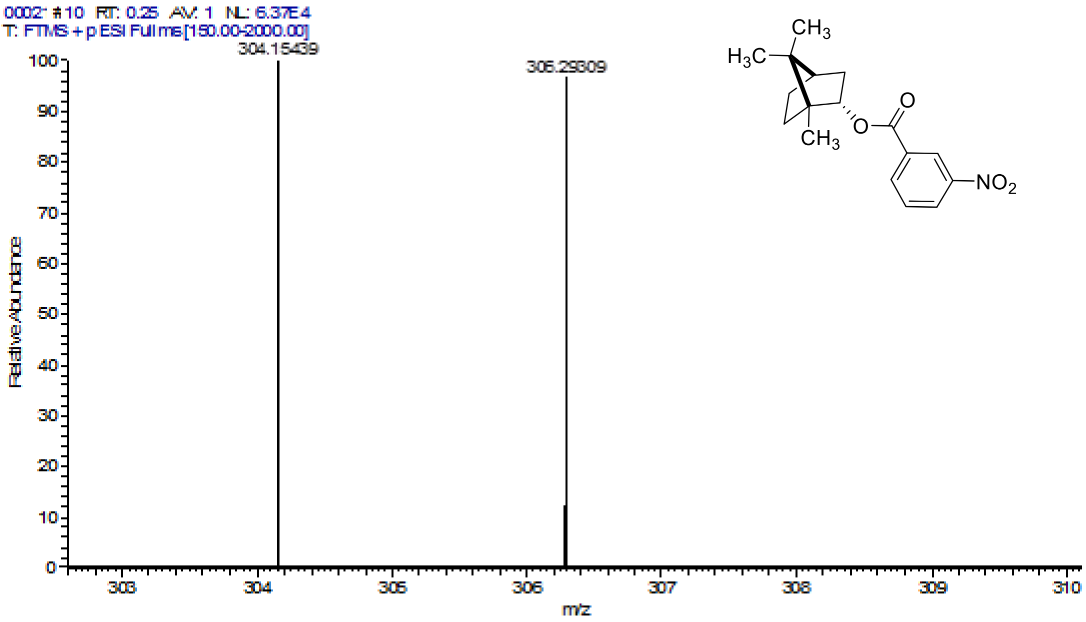

Figure 27 HRMS of **3**i

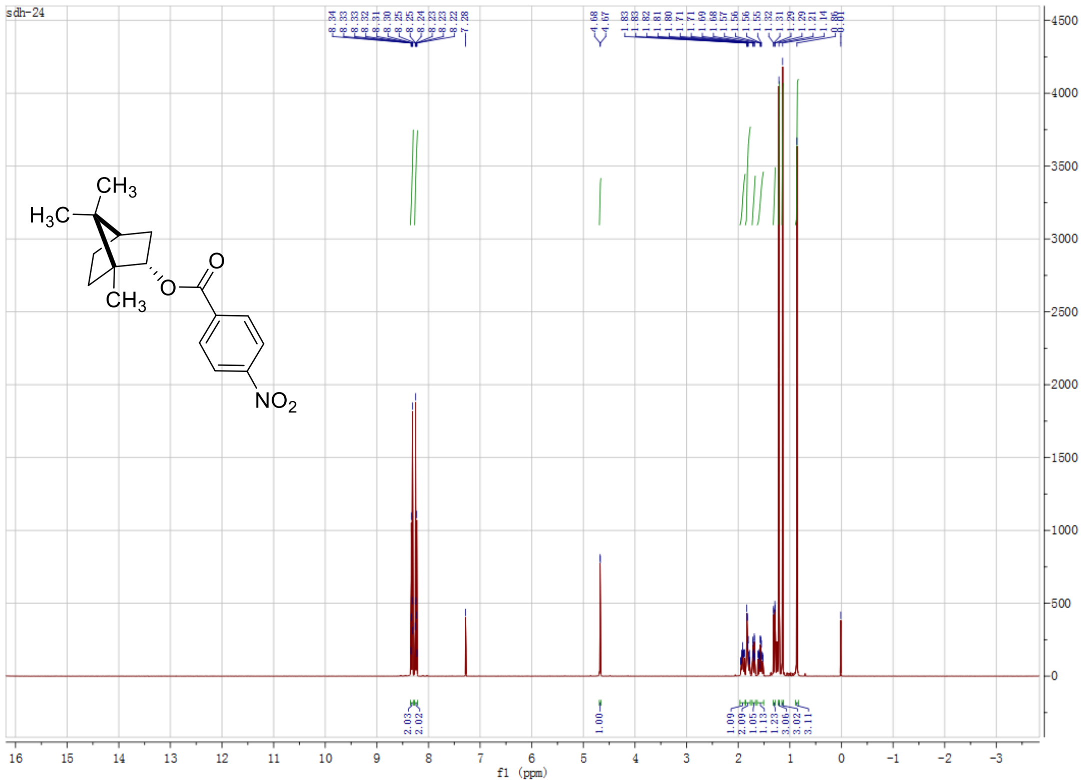

Figure 23 ^{1}H-NMR of **3**j

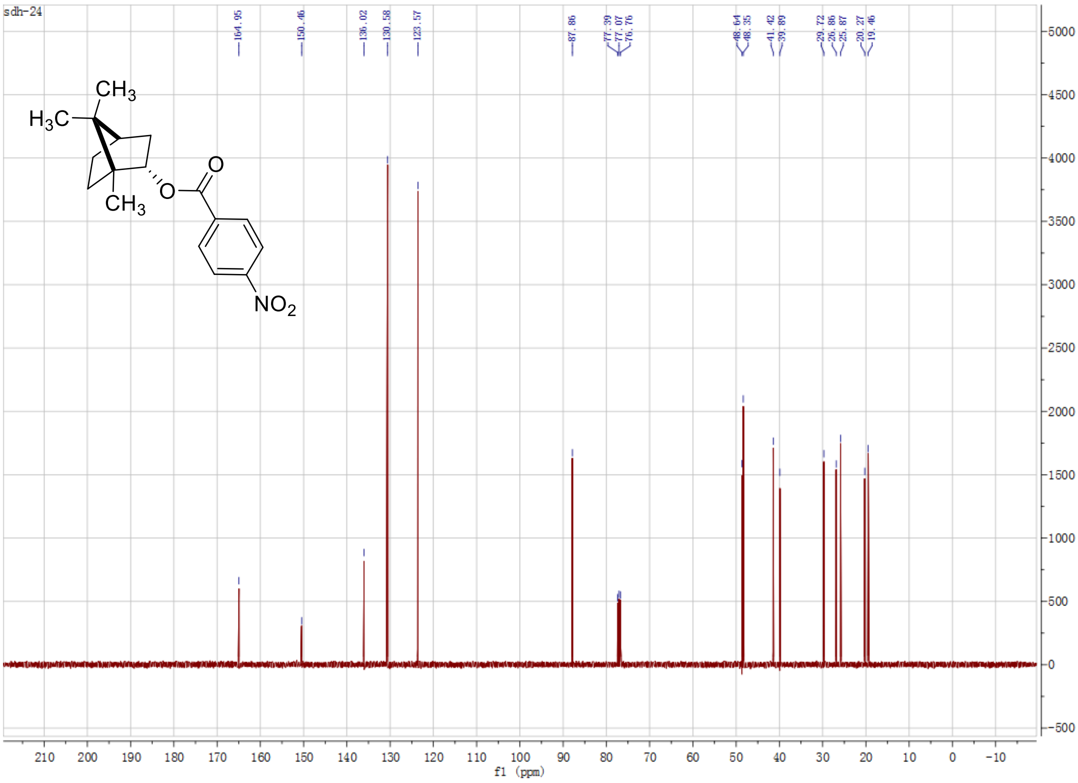

Figure 29 ^{13}C-NMR of **3**j

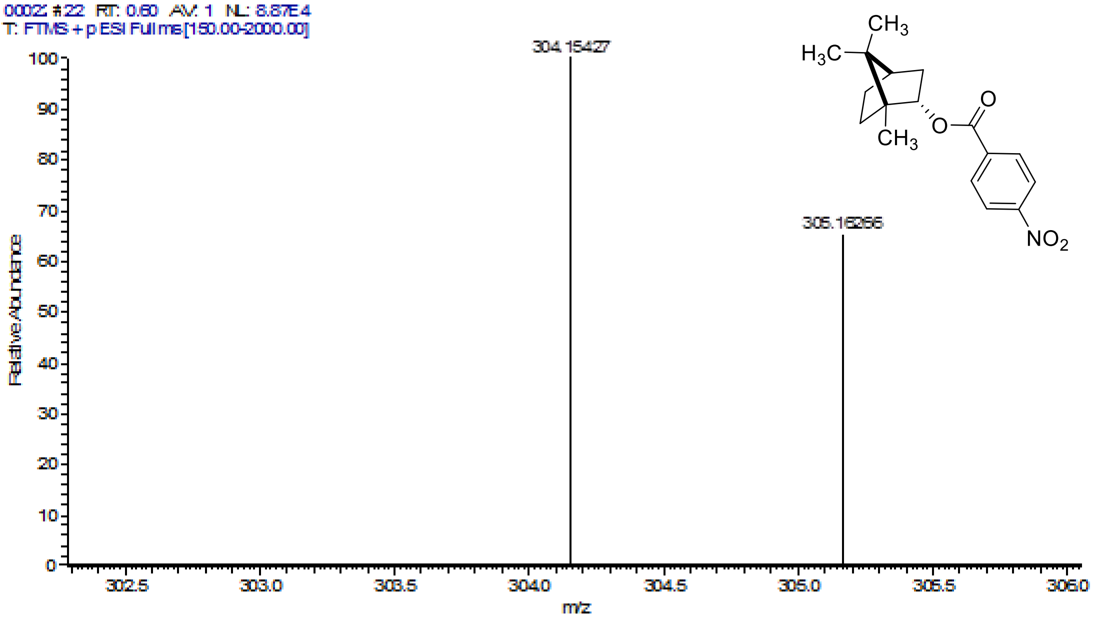

Figure 30 HRMS of **3**j

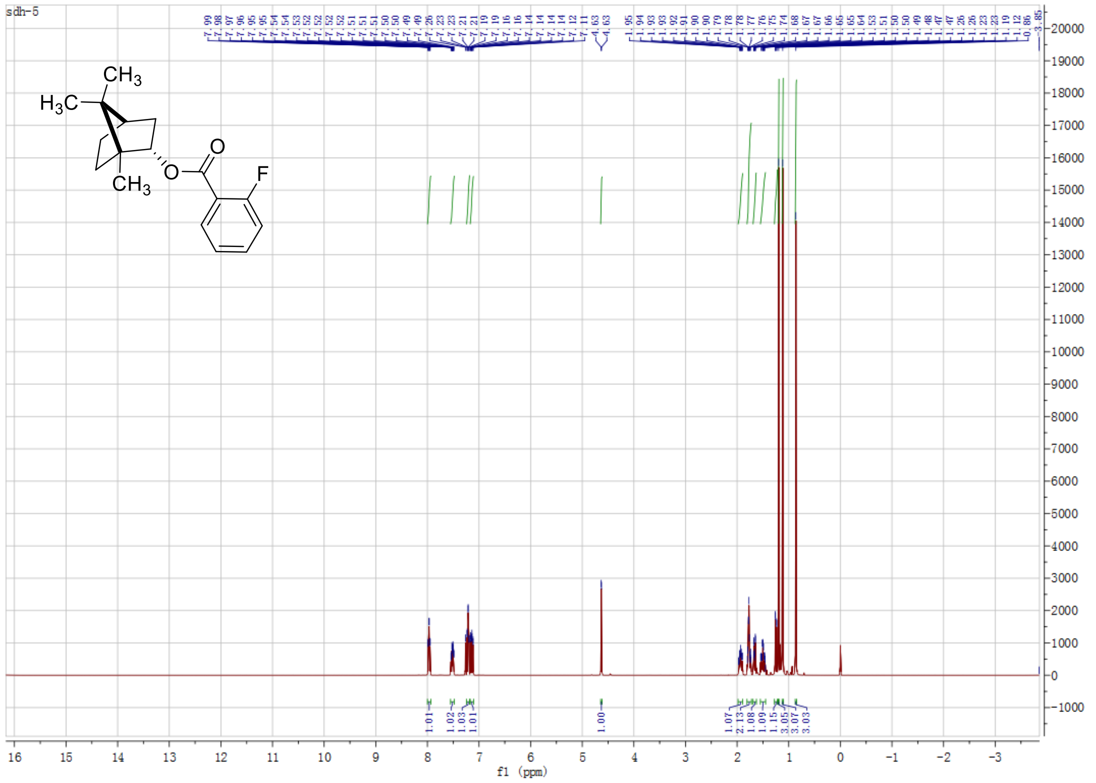

Figure 31 ^{1}H-NMR of **3**k

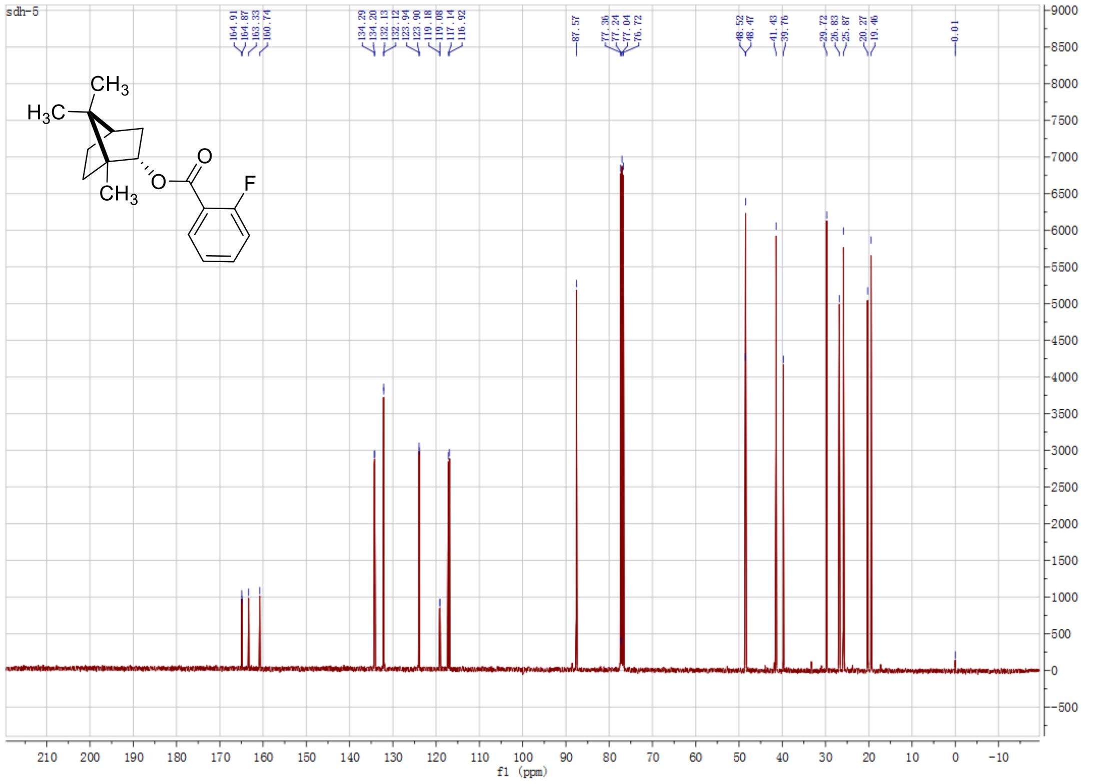

Figure 32 ^{13}C-NMR of **3**k

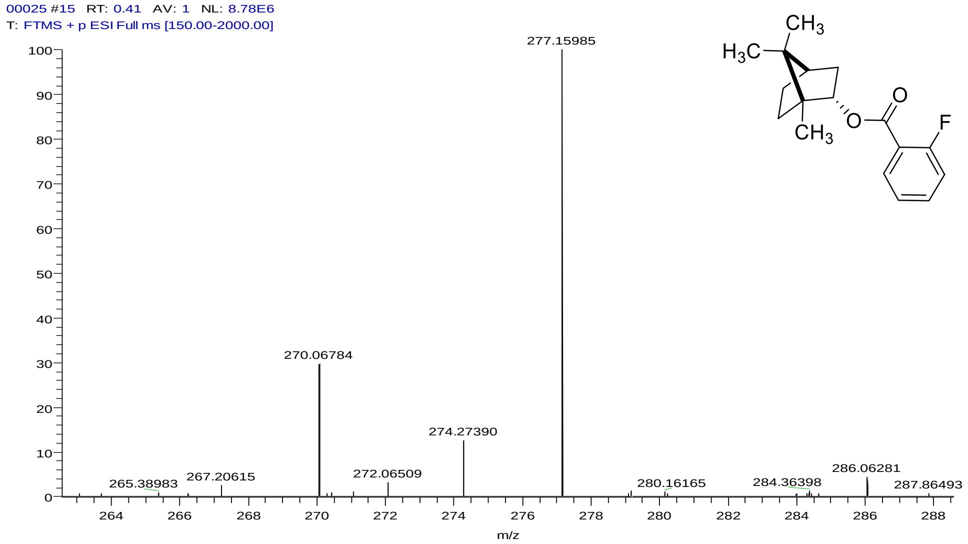

Figure 33 HRMS of **3**k

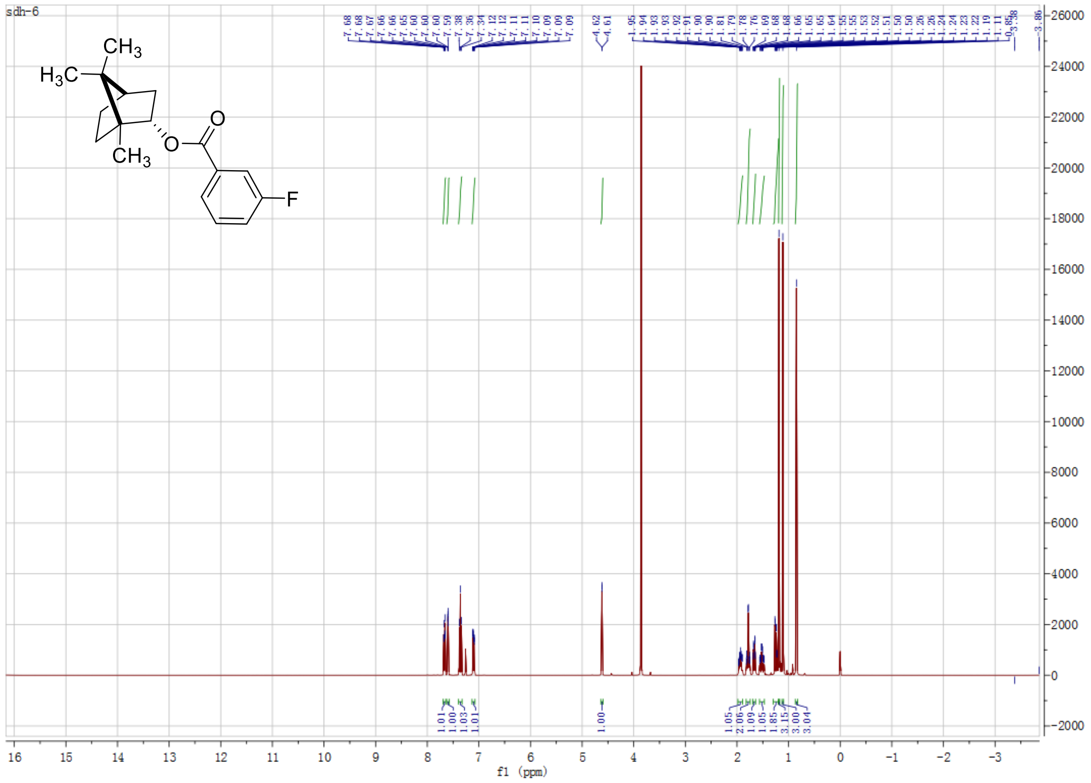

Figure 34 ^{1}H-NMR of **3**l

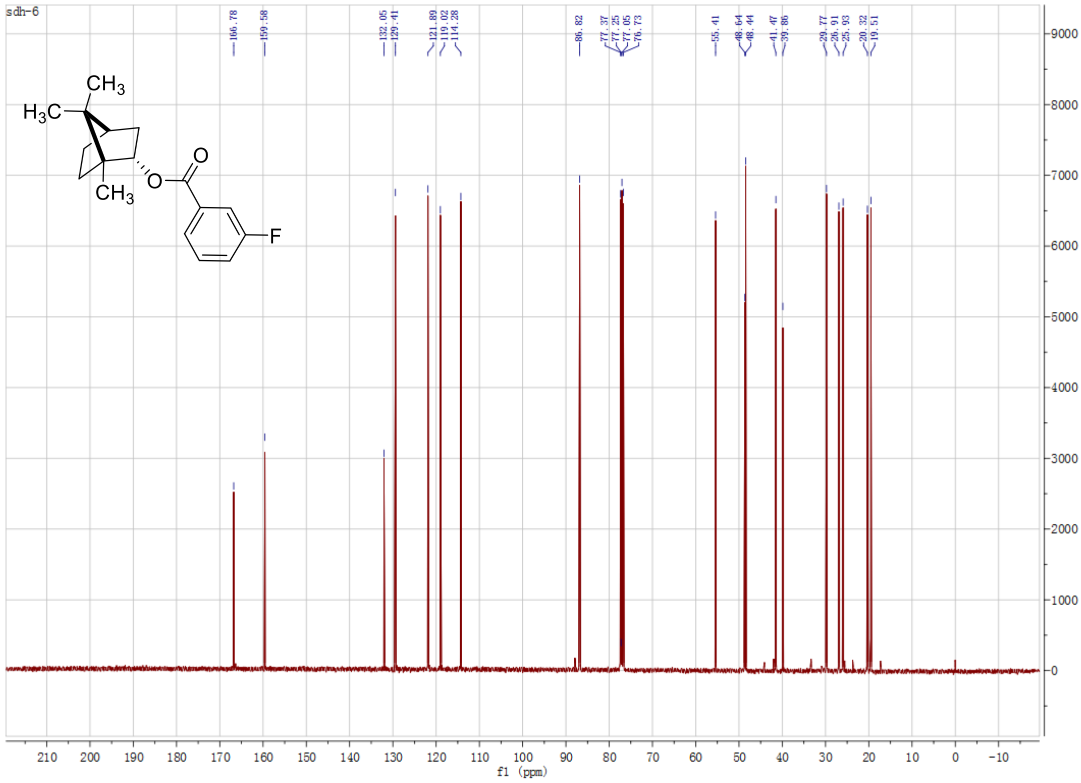

Figure 35 ^{13}C-NMR of **3**l

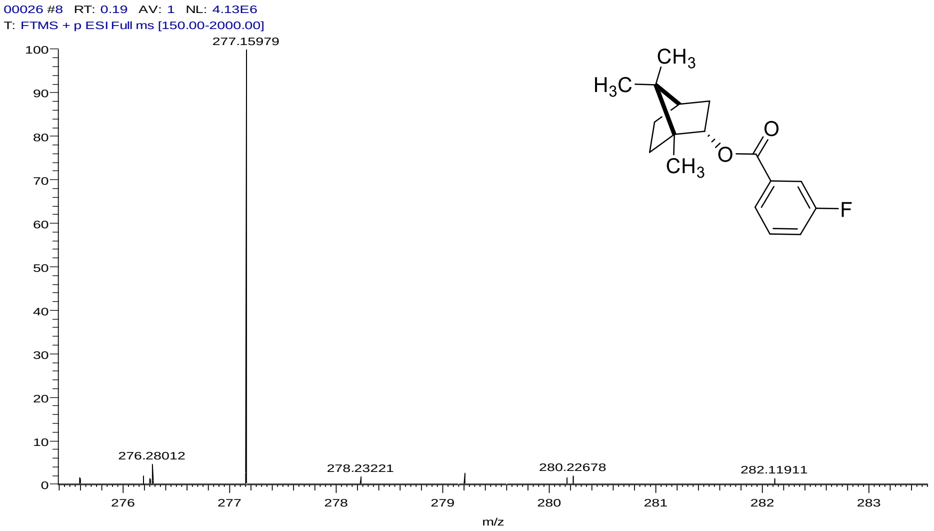

Figure 36 HRMS of **3**l

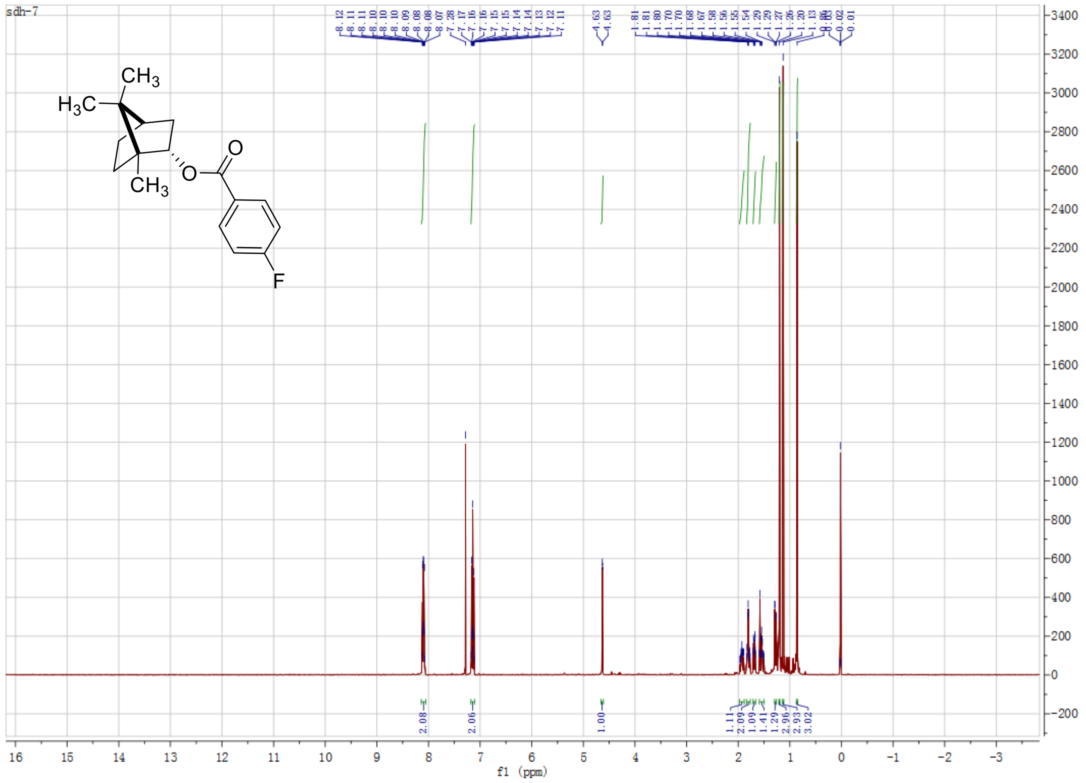

Figure 37 ^{1}H-NMR of **3**m

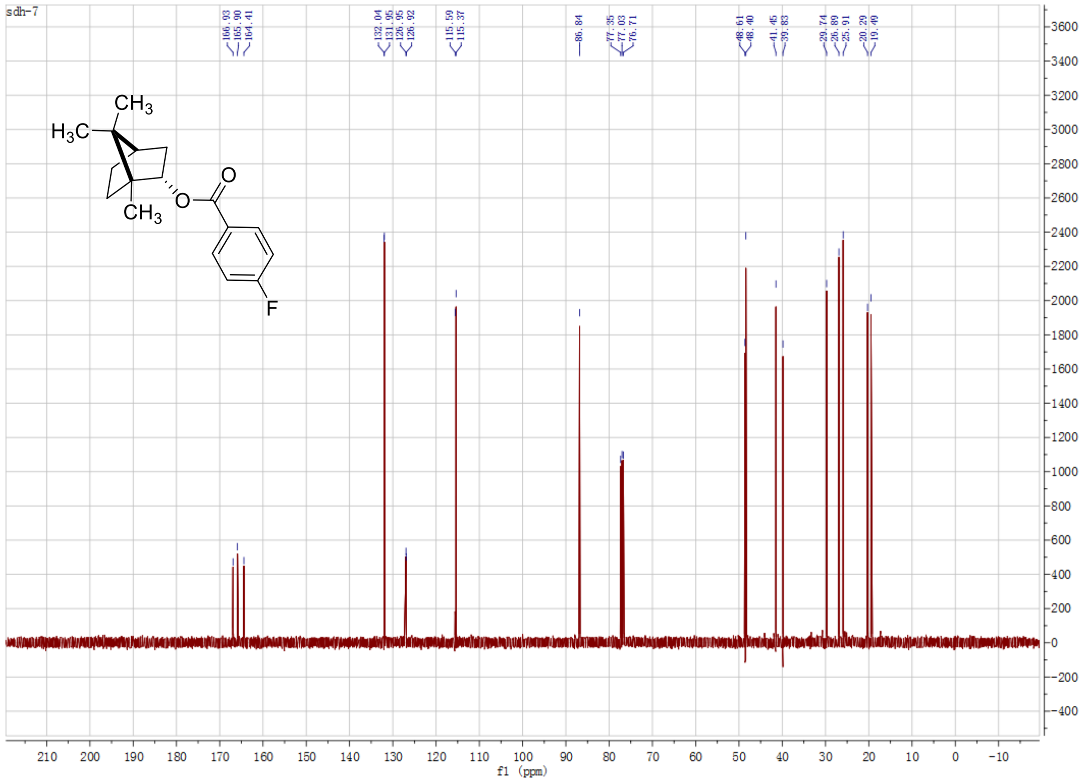

Figure 33 ^{13}C-NMR of **3**m

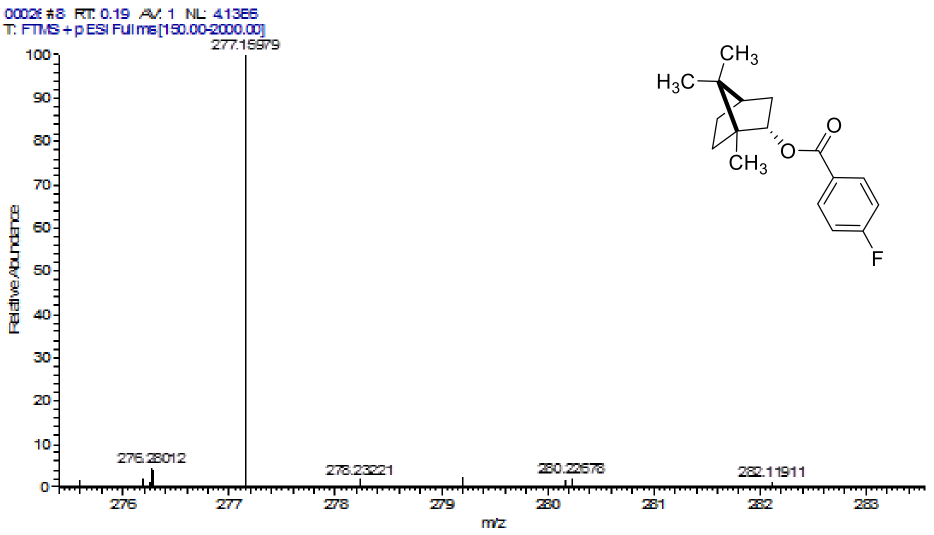

Figure 39 HRMS of **3**m

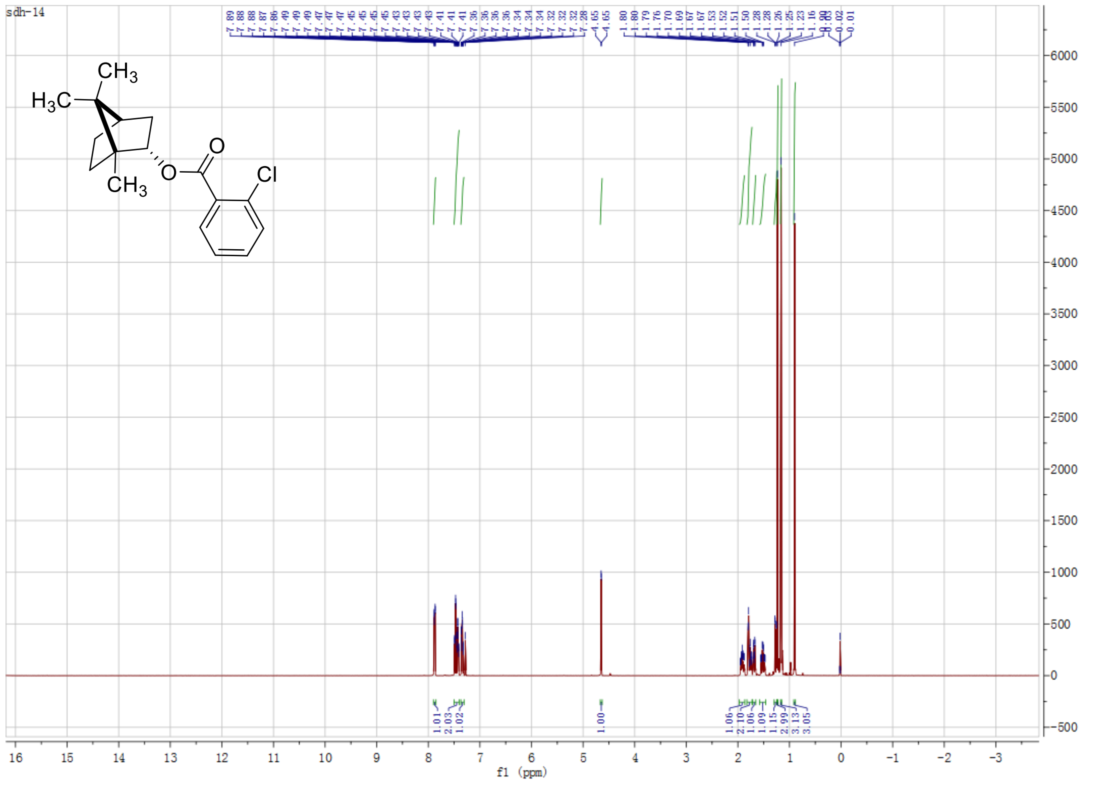

Figure 40 ^{1}H-NMR of **3**n

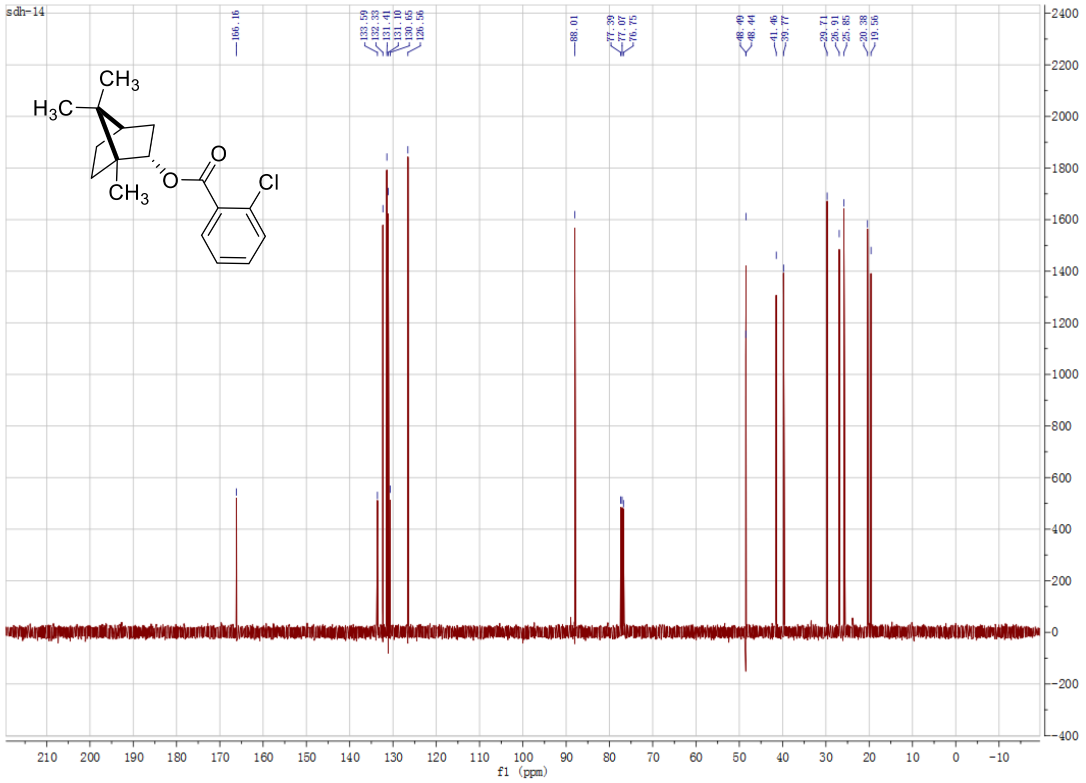

Figure 41 ^{13}C-NMR of **3**n

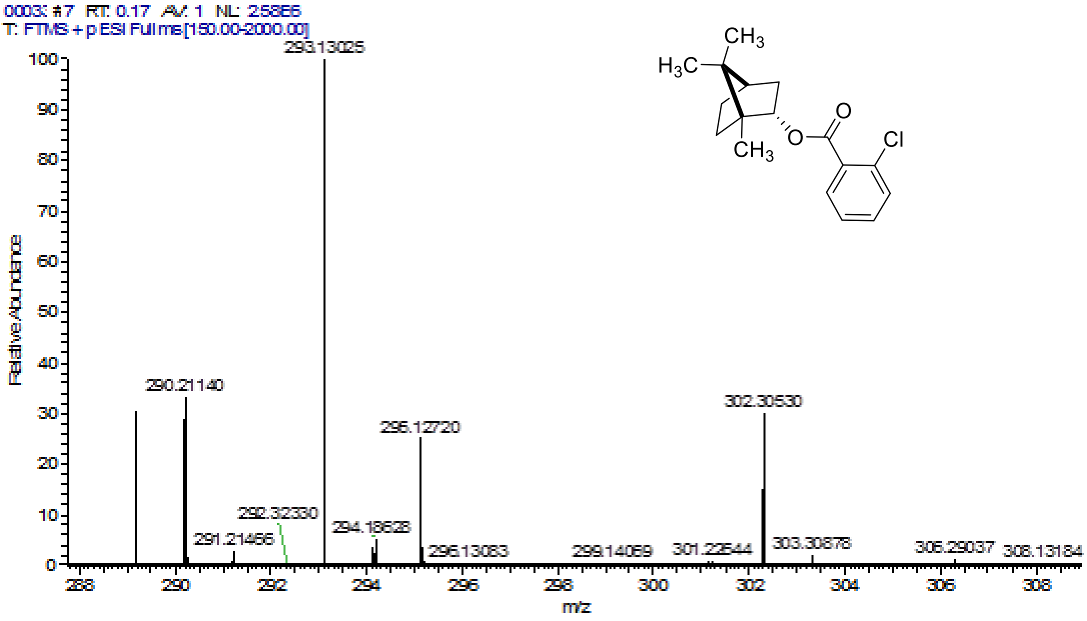

Figure 42 HRMS of **3**n

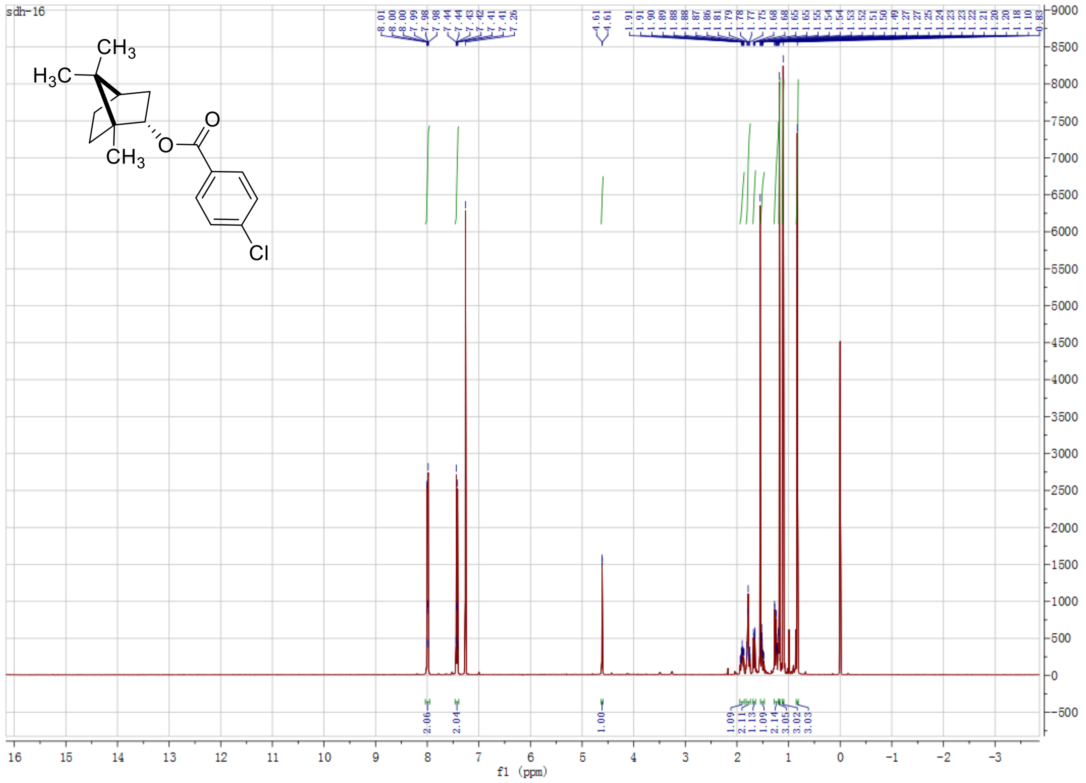

Figure 43 ^{1}H-NMR of **3**o

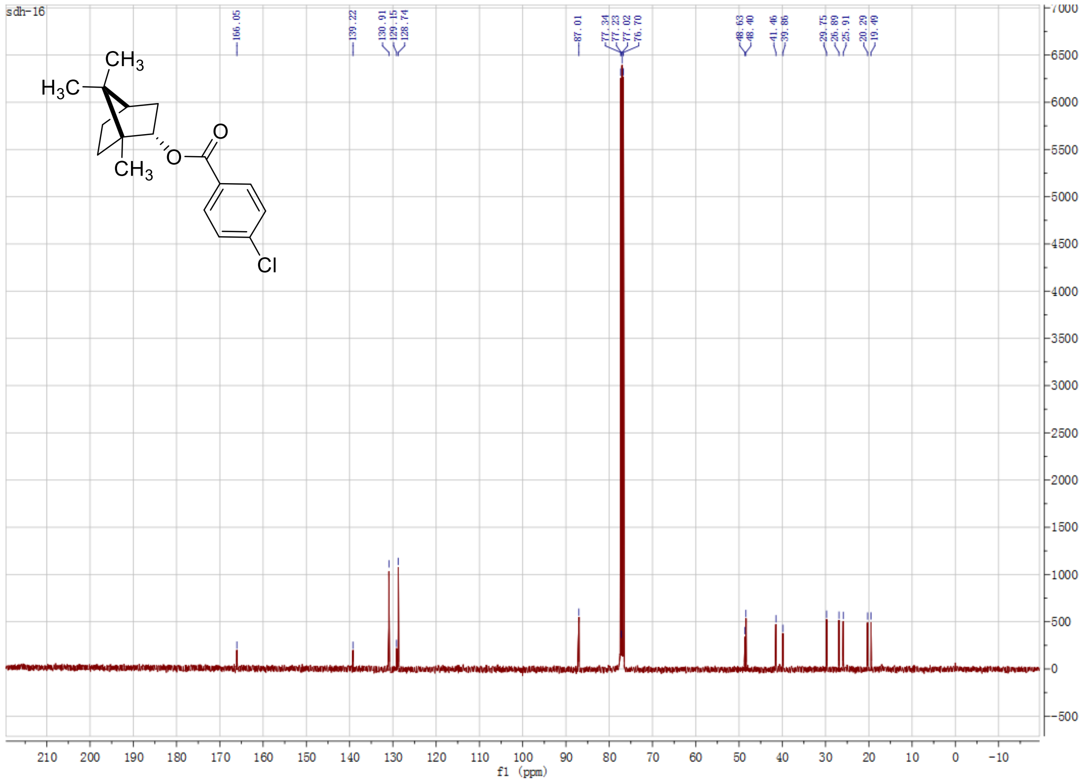

Figure 44 ^{13}C-NMR of **3**o

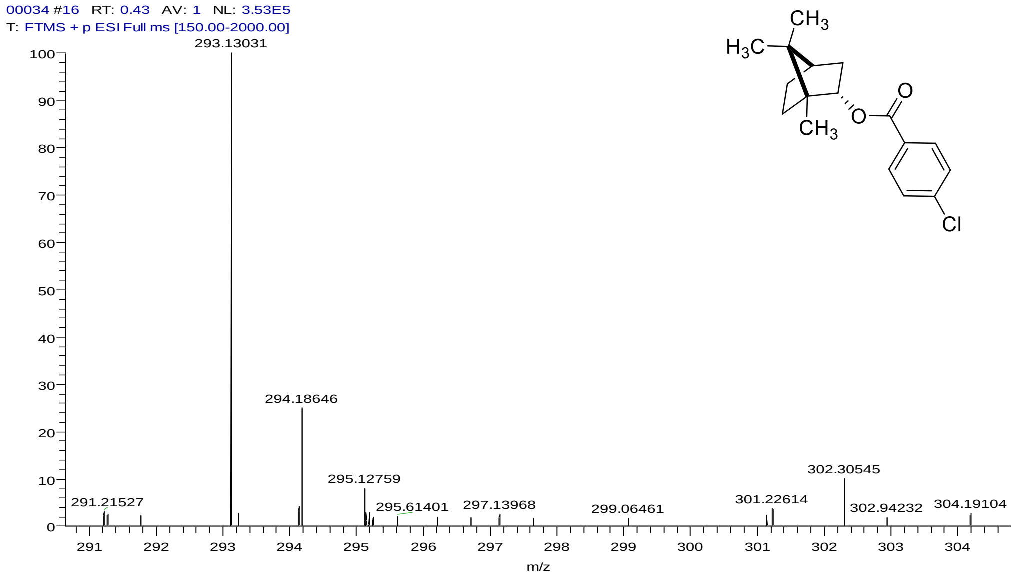

Figure 45 HRMS of **3**o

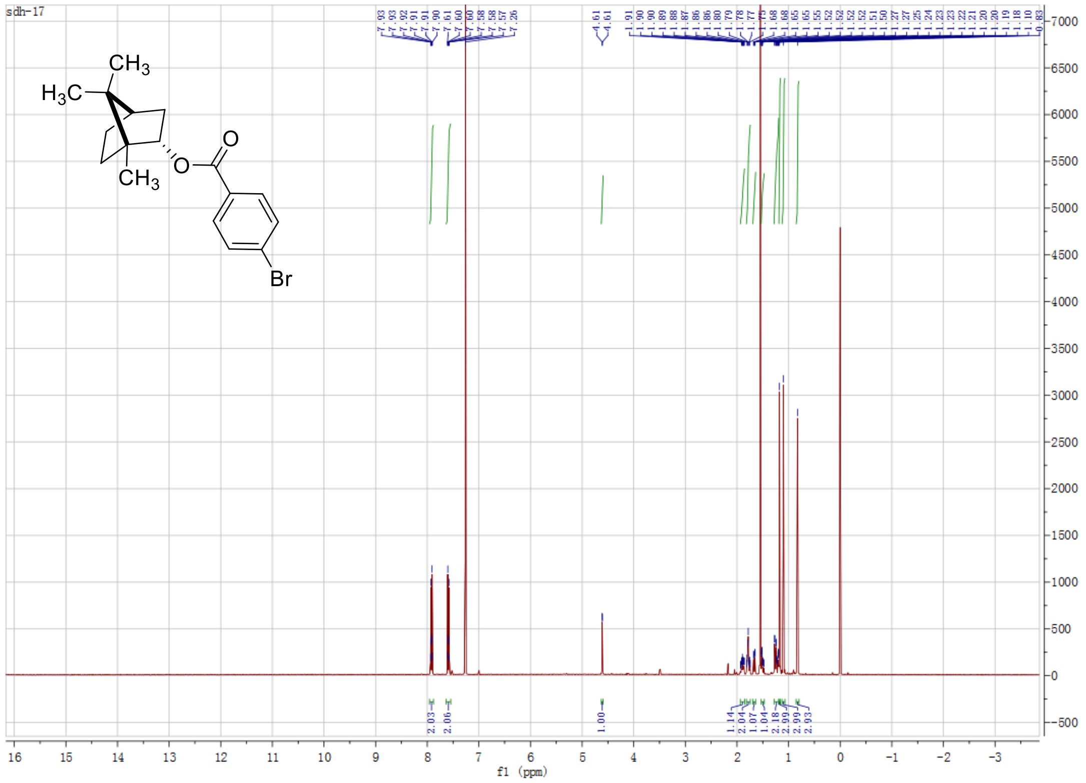

Figure 46 ^{1}H-NMR of **3**p

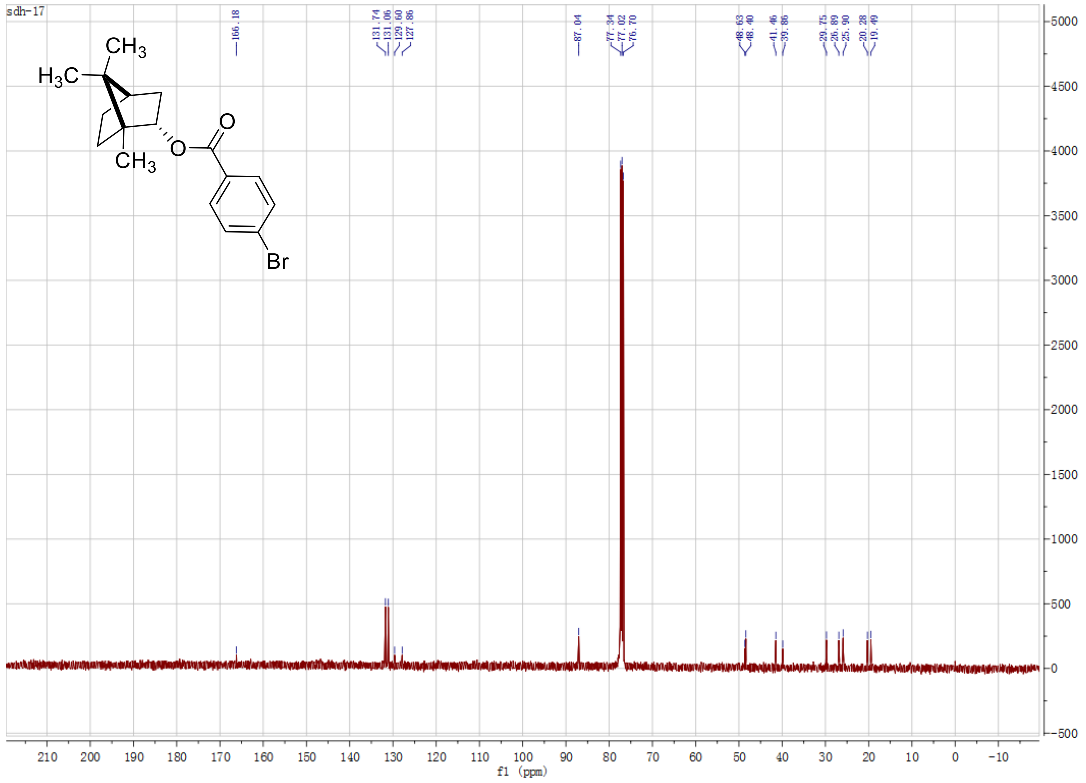

Figure 47 ^{13}C-NMR of **3**p

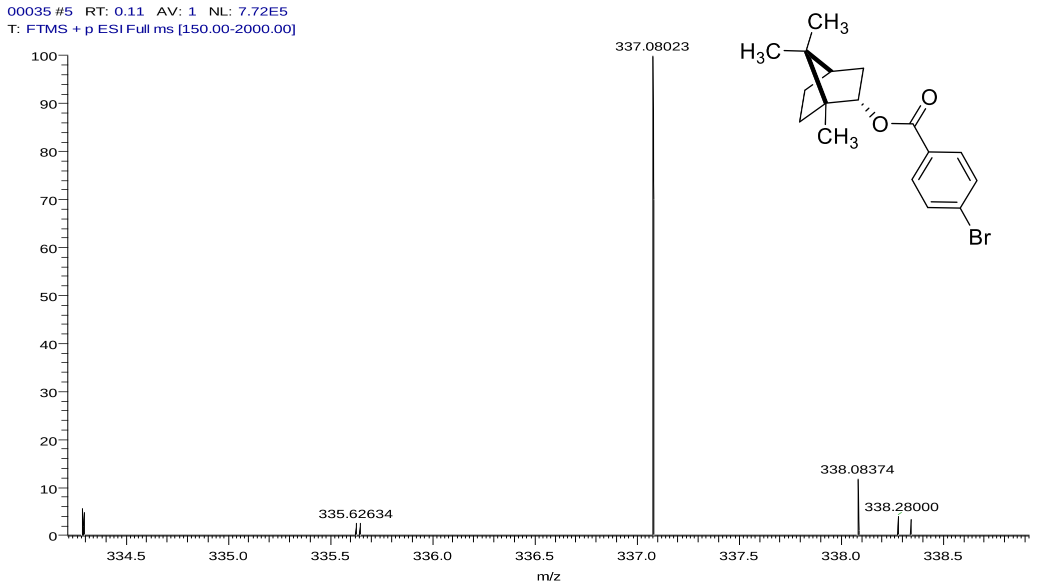

Figure 43 HRMS of **3**p

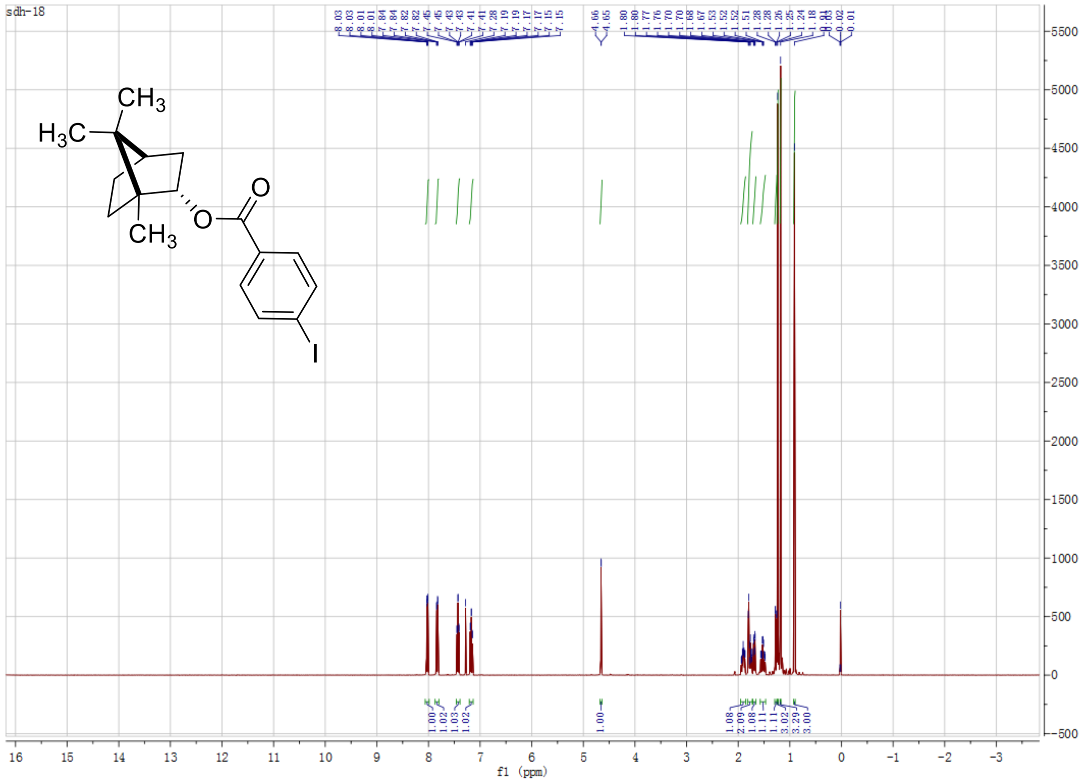

Figure 49 ^{1}H-NMR of **3**q

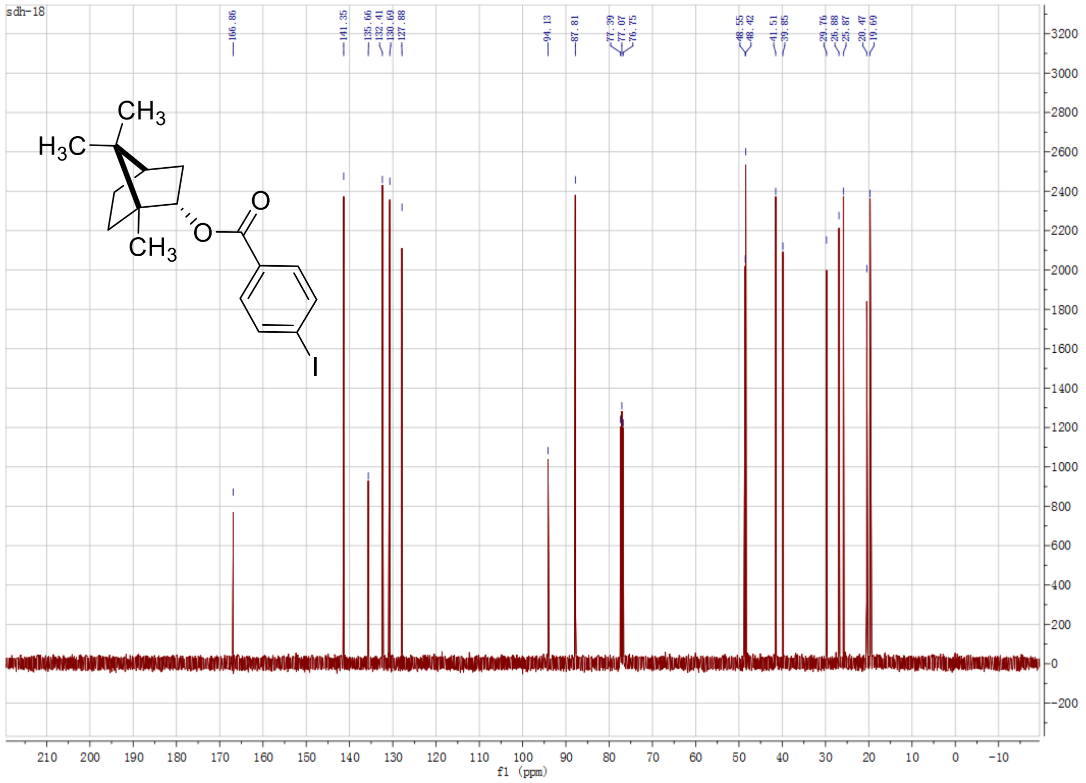

Figure 50 ^{13}C-NMR of **3**q

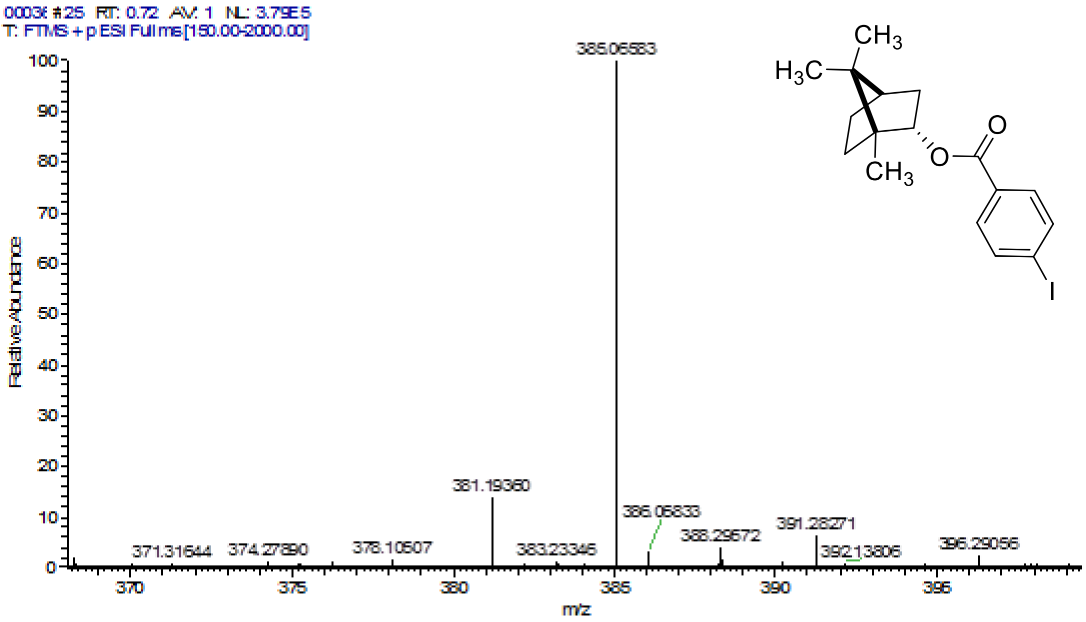

Figure 51 HRMS of **3**q

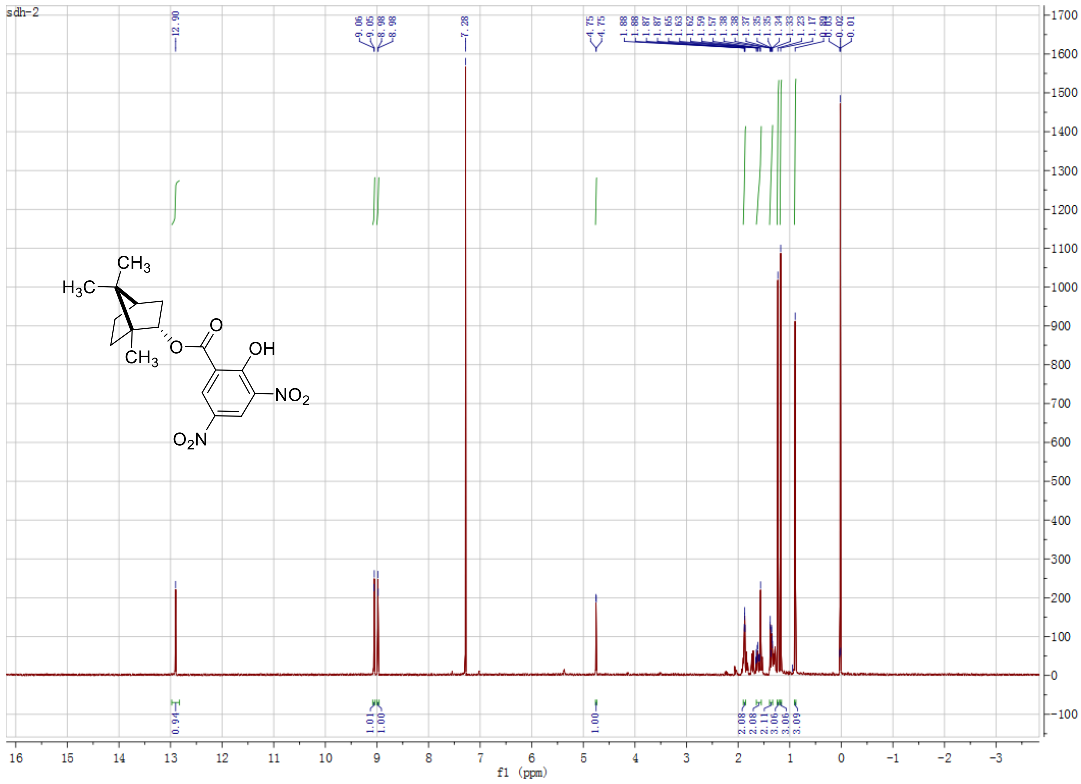

Figure 52 ^{1}H-NMR of **3**r

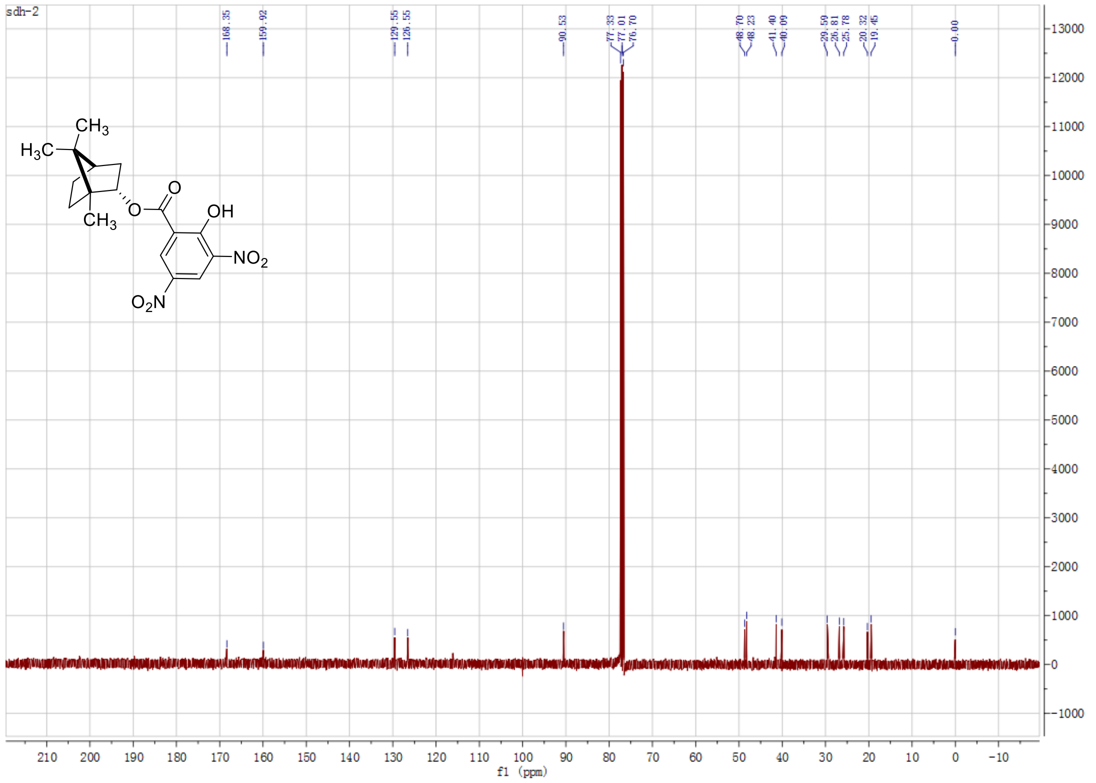

Figure 53 ^{13}C-NMR of **3r**

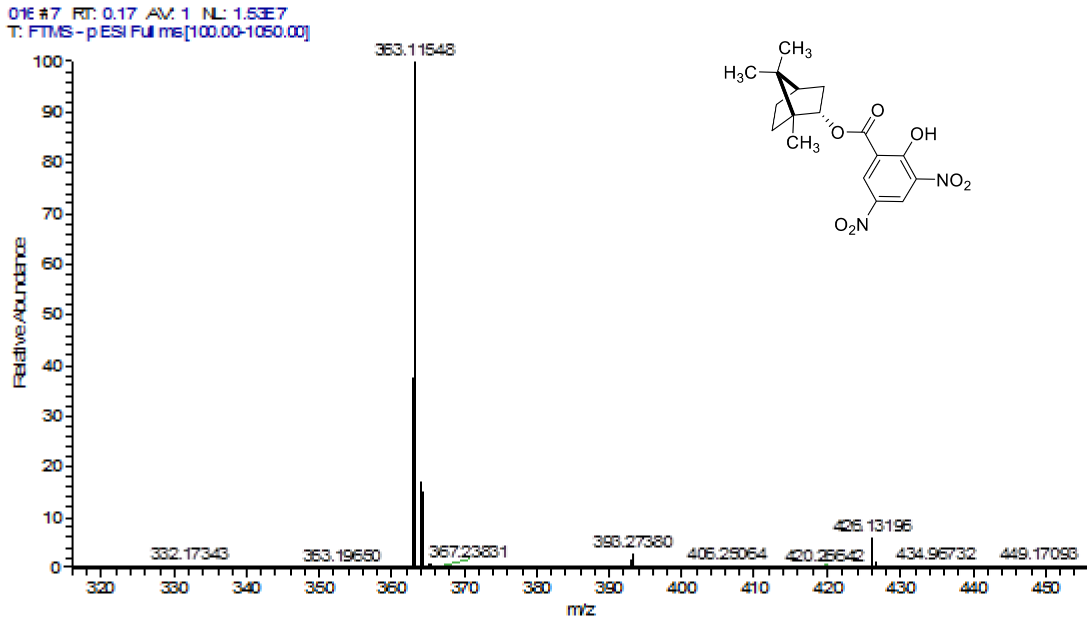

Figure 54 HRMS of **3r**

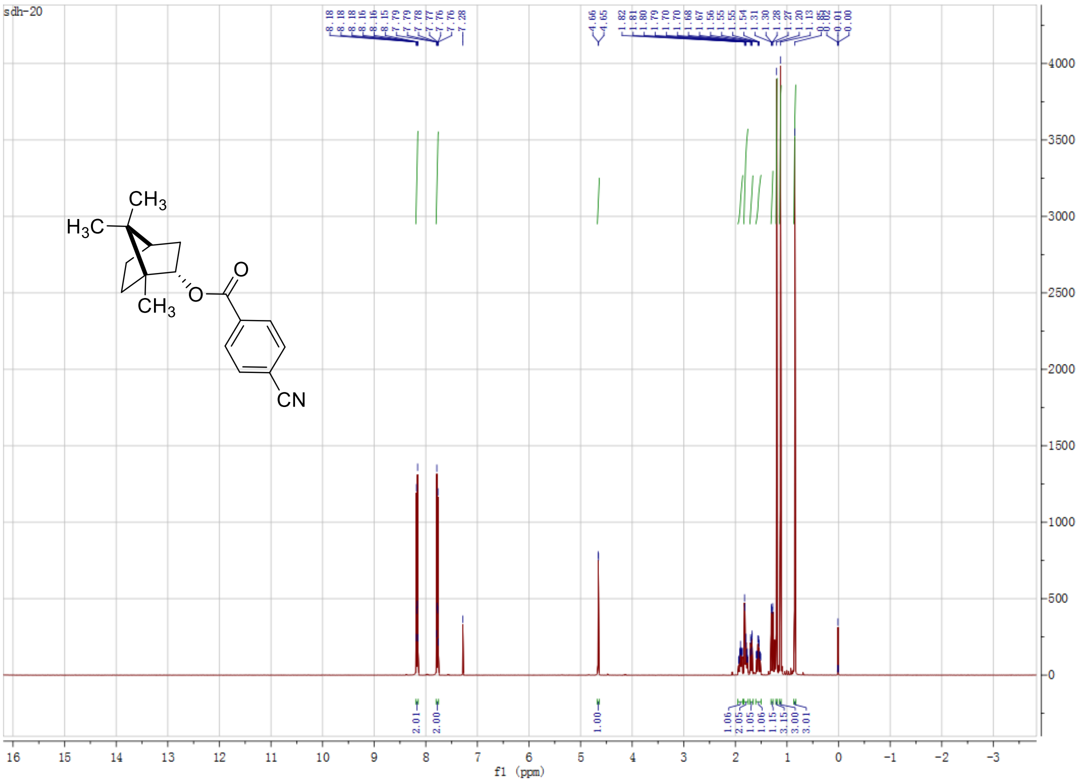

Figure 55 ^{1}H-NMR of **3**s

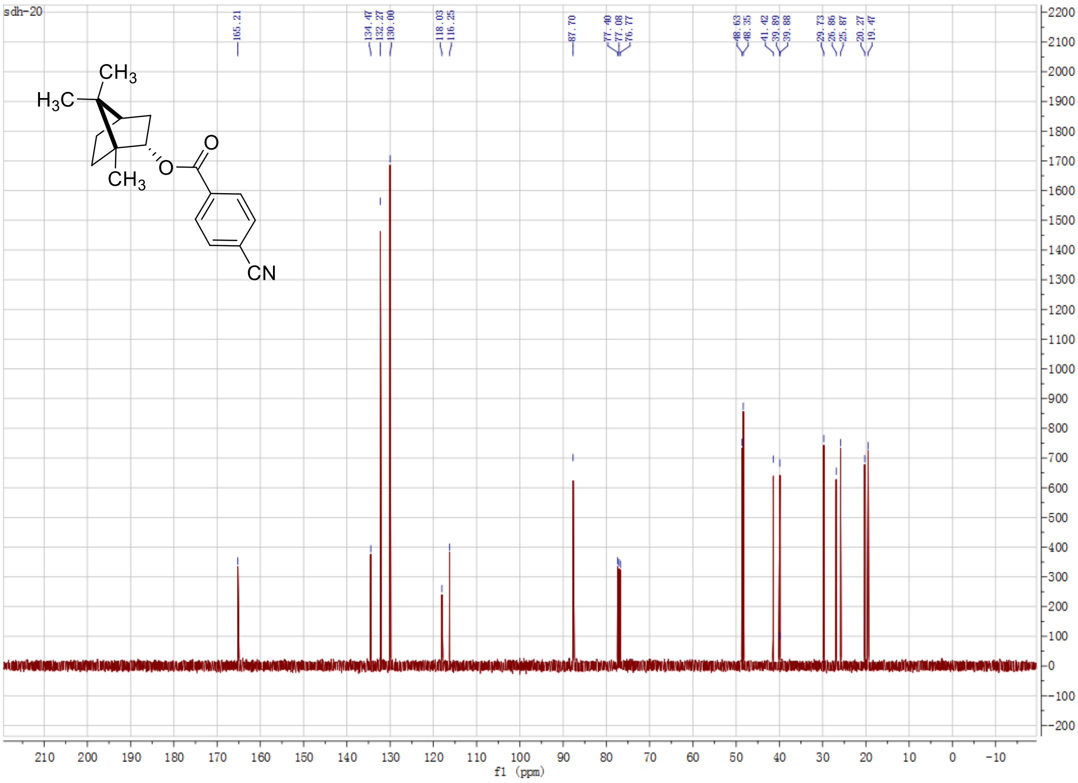

Figure 56 ^{13}C-NMR of **3**s

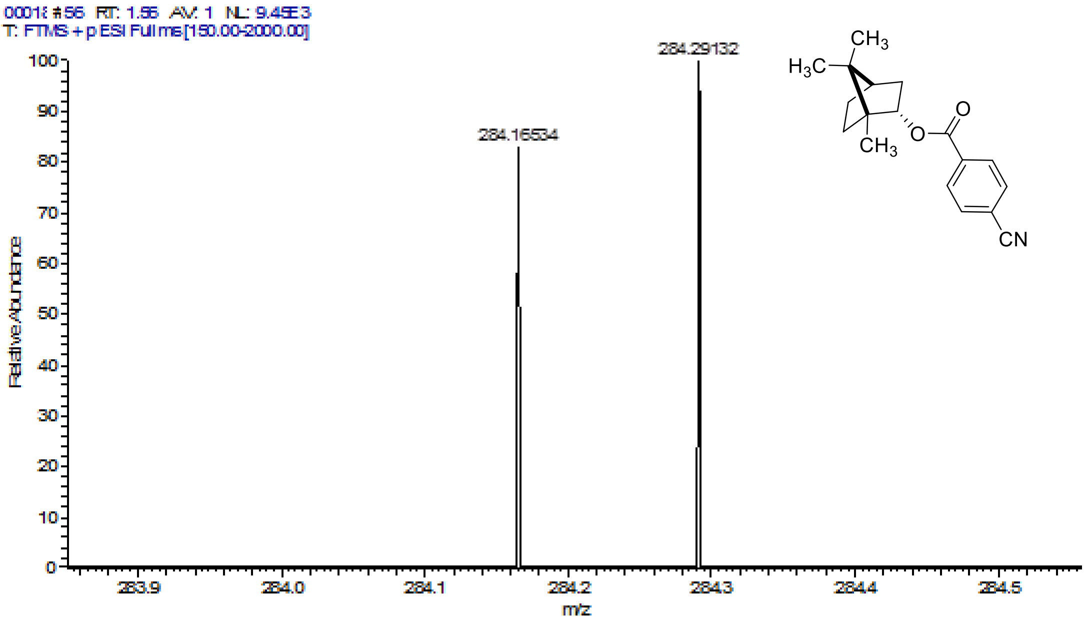

Figure 57 HRMS of **3**s

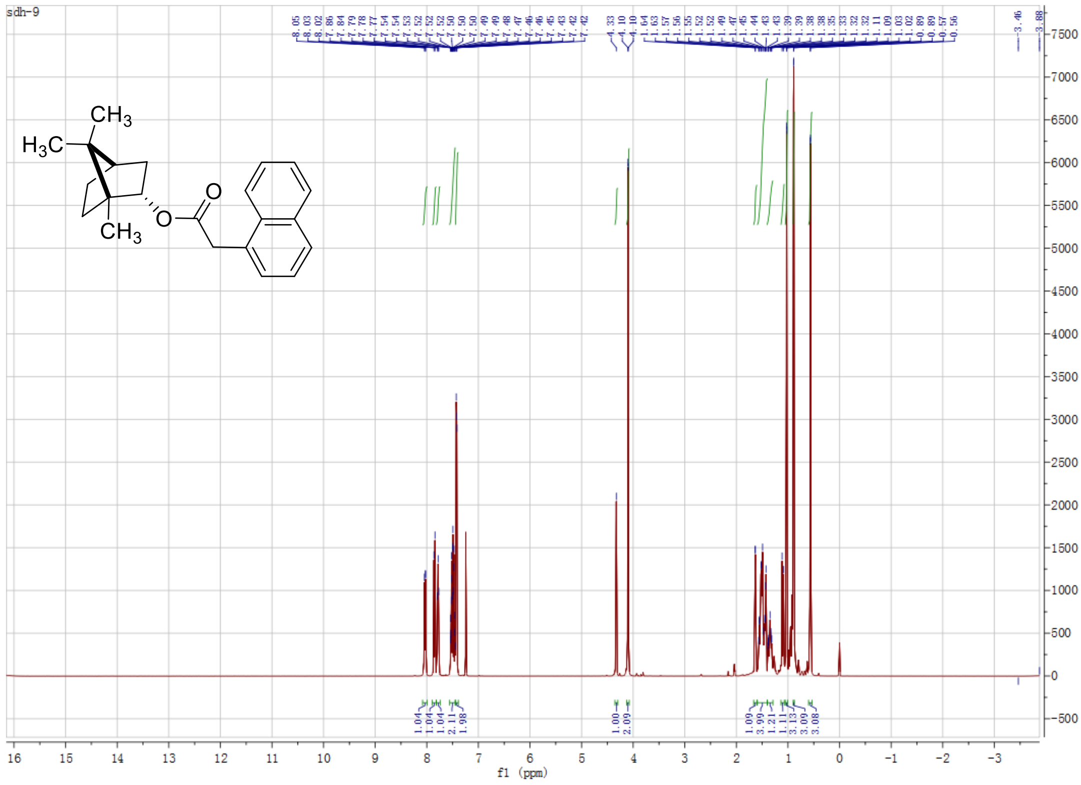

Figure 53 ^{1}H-NMR of **3**t

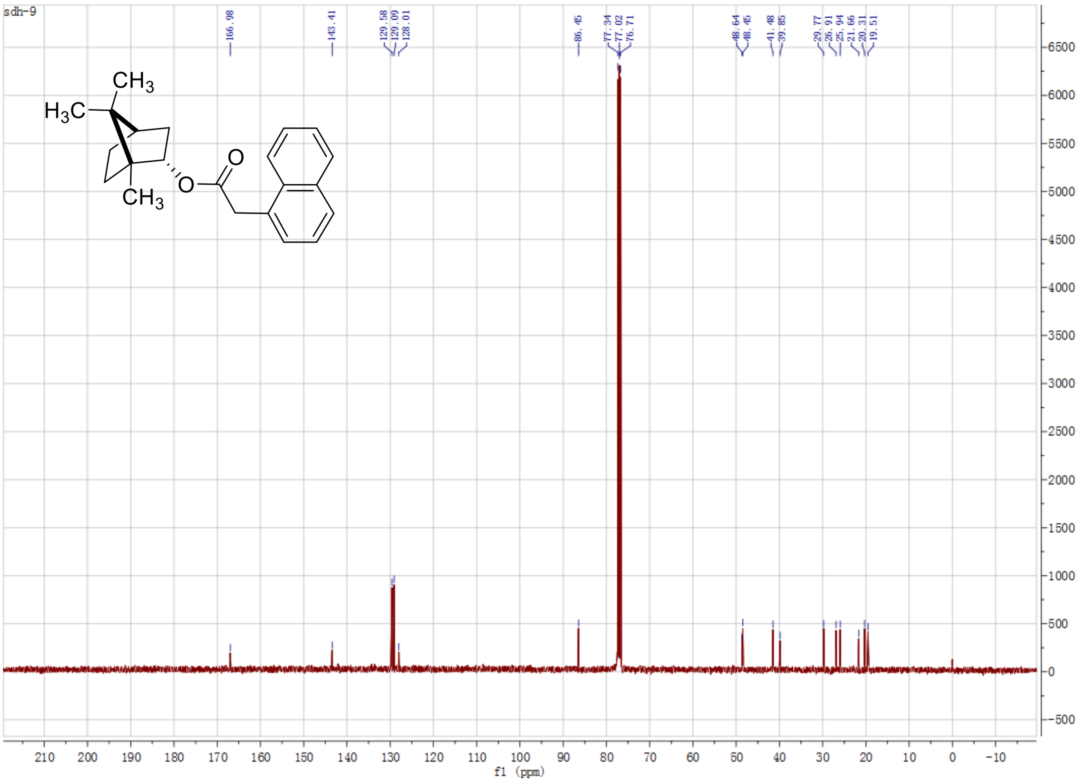

Figure 59 ^{13}C-NMR of **3**t

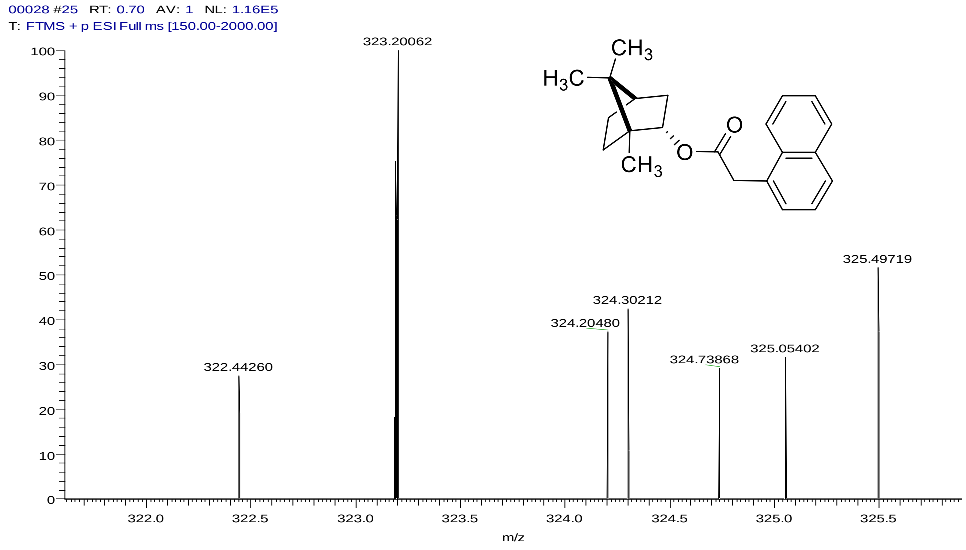

Figure 60 HRMS of **3**s
